# Edesign: Primer and Enhanced Internal Probe Design Tool for Quantitative PCR Experiments and Genotyping Assays

**DOI:** 10.1371/journal.pone.0146950

**Published:** 2016-02-10

**Authors:** Yasumasa Kimura, Takahiro Soma, Naoko Kasahara, Diane Delobel, Takeshi Hanami, Yuki Tanaka, Michiel J. L. de Hoon, Yoshihide Hayashizaki, Kengo Usui, Matthias Harbers

**Affiliations:** 1 K.K. DNAFORM, 75–1, Ono-cho, Tsurumi-ku, Yokohama, Kanagawa, 230–0046, Japan; 2 RIKEN Center for Life Science Technologies, Division of Genomic Technologies, 1-7-22 Suehiro-cho, Tsurumi-ku, Yokohama, Kanagawa, 230–0045, Japan; 3 RIKEN Omics Science Center, 1-7-22 Suehiro-cho, Tsurumi-ku, Yokohama, Kanagawa, 230–0045, Japan; 4 RIKEN Preventive Medicine & Diagnosis Innovation Program, 2–1 Hirosawa, Wako-shi, Saitama, 351–0198, Japan; University of Helsinki, FINLAND

## Abstract

Analytical PCR experiments preferably use internal probes for monitoring the amplification reaction and specific detection of the amplicon. Such internal probes have to be designed in close context with the amplification primers, and may require additional considerations for the detection of genetic variations. Here we describe Edesign, a new online and stand-alone tool for designing sets of PCR primers together with an internal probe for conducting quantitative real-time PCR (qPCR) and genotypic experiments. Edesign can be used for selecting standard DNA oligonucleotides like for instance TaqMan probes, but has been further extended with new functions and enhanced design features for Eprobes. Eprobes, with their single thiazole orange-labelled nucleotide, allow for highly sensitive genotypic assays because of their higher DNA binding affinity as compared to standard DNA oligonucleotides. Using new thermodynamic parameters, Edesign considers unique features of Eprobes during primer and probe design for establishing qPCR experiments and genotyping by melting curve analysis. Additional functions in Edesign allow probe design for effective discrimination between wild-type sequences and genetic variations either using standard DNA oligonucleotides or Eprobes. Edesign can be freely accessed online at http://www.dnaform.com/edesign2/, and the source code is available for download.

## Introduction

Many PCR applications in research and medical diagnostics monitor amplification reactions in real-time to directly detect the amplification product or to obtain quantitative results. Preferably this is achieved by adding an internal probe to the reaction that hybridizes to the amplification product in a sequence-dependent manner [1]. Using such an additional hybridization probe increases the specificity of the detection step as compared to the use of intercalating dyes such as SYBR Green that just measure the amount of DNA in the reaction mixture [2]. Furthermore, multiple internal probes can be combined in one PCR reaction to allow for the detection of different amplification products in a “multiplexed” PCR assay. Over time different types of hybridization probes were developed for quantitative real-time PCR (qPCR) assays, where most designs use fluorescence resonance energy transfer (FRET) [3] between a donor and an acceptor fluorochrome to control the signal. Typical examples for fluorescent detection probes for qPCR experiments are TaqMan probes [4], which are probably the most commonly used probes, and Molecular Beacons [5]. In addition, two separate oligonucleotides have been used as so-called HybProbes [6], where one oligonucleotide carries a donor and the other oligonucleotide carries an acceptor dye. All those internal probes can only be designed in the context of the primers that are used in the amplification reaction. However, while different computational tools are available for designing PCR primers and characterization of their features [7–12], only very few tools like Primer3 are in the public domain that allow for automated design of an internal probe. Within Primer3 [7,8], a popular primer design tool, probe design features are limited, and the software does not calculate the specificity of an internal probe and possible complementarities between an internal probe and the primers. Therefore many laboratories are also using commercial applications offered by different providers; for a list of software tools refer to [13].

While standard internal probe designs work well for regular qPCR experiments, new challenges arise from needs to develop reliable genotyping assays. Large-scale sequencing of various genomes provides detailed information on genetic variation, and for the human genome further details on disease-related mutations are available. To make use of this information for medical diagnostics or genotyping experiments, highly reliable detection methods have to be developed that allow for fast assay development on a high throughput basis. One approach to address this need is the combination of PCR amplification with melting curve analysis of the amplification products. New high-resolution melting curve methods [14–16] can distinguish mutations even in a single base pair, when using suitable internal probes. Desirable for such assays are short probes with a higher binding affinity to the opposite strand than standard DNA oligonucleotides, which can only be obtained by changing chemical features of a DNA oligonucleotide or by using special intercalators like intercalating dyes.

**Fig 1:**
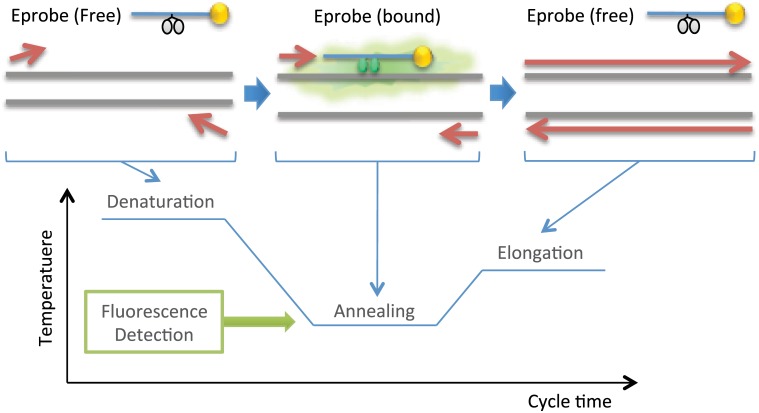
Mechanism of Eprobe mediated PCR. During the denaturation step of a PCR reaction, an Eprobe is single-stranded and its signal is suppressed by an excitonic interaction between the two dye moieties (indicated in grey). During the annealing step, the Eprobe binds to its target sequence, and therefore emits a strong fluorescent signal (indicated in green). In qPCR experiments, an Eprobe-derived fluorescent signal is detected during the annealing step. Eprobes are designed in such a way that they commonly have a *T*_M_ below the elongation temperature. Therefore an Eprobe is normally single-stranded during the elongation step and hence does not interfere with the polymerase extension reaction. As indicated in yellow, the 3’ end of an Eprobe is blocked to avoid any primer extension reactions from the probe.

As an alternative to FRET-based probes, we recently developed Eprobes (Fig 1), a new detection method for qPCR assays and mutation detection in standard [17,18] and high-resolution melting curve experiments [18]. Eprobes are 3’-end-blocked Exciton-Controlled Hybridization-sensitive fluorescent Oligonucleotides (ECHOs) that require for signal generation only one labelled nucleotide with two covalently attached dye moieties [19,20]. The signal of an ECHO is strongly suppressed in the single stranded state by an excitonic interaction between the two dye moieties. The excitonic interaction between the dyes is disrupted after hybridization to a complementary DNA strand, leading to a strong fluorescence signal from the dye moieties that interact now with neighboring nucleotides in the double-stranded oligonucleotide. Presently, thymidine linked thiazole orange is the most preferable dye for designing Eprobes, although other dyes have been reported for the preparation of ECHOs [20,21] and Eprobes [17]. Our analysis of the thermodynamic features of thiazole orange-labelled ECHOs showed a strong enhancement of the binding affinity of those ECHOs as compared to standard DNA oligonucleotides [22]. Therefore Eprobes are an excellent choice for mutation detection assays using melting curve analysis, but better computational design methods are needed to assist their use on a large scale, and to take into account the special features of Eprobes compared to other hybridization probes.

Here we describe Edesign, a new freely accessible online and stand-alone tool for the design of qPCR experiments with internal probes. For genotyping, Edesign has additional functions implemented for effective discrimination of target variants by internal probes. To fully implement the design of Eprobes into Edesign, we further investigated positional effects of the labelled nucleotide and mismatched base pairs in ECHO/DNA duplexes. In this way, we addressed the specific features of Eprobes as compared to working with standard DNA oligonucleotides such as TaqMan probes in PCR experiments. We further provide a tool for future updates of Edesign for other types of internal probes making Edesign an extendable PCR design platform.

## Materials and Methods

### Materials

High-performance liquid chromatography-purified ECHOs (product name “Eprimer”) and Eprobes were purchased from DNAFORM K.K. (Yokohama, Japan), and high-performance liquid chromatography-purified standard DNA oligonucleotides were purchased from SIGMA-ALDRICH Japan K.K. (Tokyo, Japan).

### Sequence design

A reference sequence for testing positional effects was selected from randomly generated 20-mer sequences with the goal to avoid any strong secondary structures and to contain a thymidine at every second position. Based on sequence 5’-TGTGTATCTTTCTCTTTCTC-3’ 10 ECHOs were prepared having a thiazole orange-labelled thymidine in the 2^nd^, 4^th^, 6^th^, 8^th^, 10^th^, 12^th^, 14^th^, 16^th^, 18^th^ or 20^th^ position respectively. For melting curve analysis, one perfectly complementary DNA oligonucleotide was made along with 60 antisense DNA oligonucleotides that cover all possible single base mismatches in the 20-mer sequences. In addition, ECHOs 5’-TTTATCGTTCGCTTT-3’ and 5’-TTTCCTACCCACTTTTCTCCCATTT-3’ were designed by the same approach to confirm positioning effects for thiazole orange-labelled thymidine in the end positions.

ECHOs SELF_A: 5’-ACTTTTTTGCATTAGCAAAT-3’ and SELF_B: 5’-ACTTTCGTTTTTTTAAACGT-3’ were selected to form stem loop structures with thymidines positioned within the single-stranded region, stem structure and the hairpin region.

Thermodynamic parameters for the nearest neighbor effects for mismatches next to a thiazole orange-labelled thymidine were analyzed by a set of 24 ECHOs listed in S2 Table.

### Melting curve experiments

Melting curve experiments with real-time fluorescent monitoring were performed as described in a previous study [22]. In brief, fluorescence melting curves were measured at 530 nm by scanning the temperature range from 4 to 95°C. The thermodynamic parameters of each fluorescence melting curve were calculated and then averaged for each set of oligonucleotides using three independent measurements. The fluorescence values at 20, 40 and 60°C were taken from the melting curves as representative data points.

### Melting curve data analysis

The melting curves *Y* measured by the logarithmic fluorescence at 530 nm were analyzed using a two-state thermodynamic model. Enthalpy Δ*H°* and entropy Δ*S°* were obtained through curve-fitting of the two-state model equation to the measured melting curve *Y* as described in the previous study [22]. With the fitted values for Δ*H°*, Δ*S°*, the Gibbs free energy at temperature *T* Δ*G°*_*T*_ is given by

$$\Delta G_{T}^{o}=\Delta H^{o}-\left(T+273.15\right)\Delta S^{o}$$

The melting temperature *T*_M_ for duplex formed by a couple of complementary oligonucleotides is given by

$$T_{\text{M}}=\frac{\Delta H^{o}}{\Delta S^{o}+R\text{ln}\left(\frac{C_{T}}{4}\right)}-273.15$$

where *C*_T_ is the total single-strand concentration.

*T*_M_ for self-folding of single oligonucleotide is given by

$$T_{\text{M}}=\frac{\Delta H^{o}}{\Delta S^{o}+R\text{ln}C_{T}}-273.15$$

Negative first derivative of the melting curve *W* was calculated as the averaged negative slope of *Y*(*T*_*i*_) using five data points around a particular temperature *T*_*i*_.

$$W\left(T_{i}\right)=-\frac{1}{5}\overset{2}{\underset{d=-2}{\sum }}\frac{Y\left(T_{i-d}\right)-Y\left(T_{i-d-1}\right)}{T_{i-d}-T_{i-d-1}}$$

Curve fitting and other calculations described here were performed by using the statistical computing environment R version 2.12 [23].

### Calculation of incremental thermodynamic parameters

As in the previous study [22], we assumed that the thermodynamic parameters of ECHO/DNA duplexes are comprised of a contribution of the DNA/DNA duplex and an incremental effect by the thymidine covalently labelled with two thiazole orange moieties (T^E^). Accordingly, the enthalpy of an ECHO/DNA duplex is given by

$$\Delta H_{\left(\text{ECHO}/\text{DNA}\right)}^{o}=\Delta H_{\left(\text{DNA}/\text{DNA}\right)}^{o}\;+\Delta \Delta H^{o}$$

The incremental thermodynamic parameters of a T^E^ substitution, ΔΔ*H°*, ΔΔ*S°*, ΔΔ*G°*_37_ and ΔΔ*G°*_60_ were calculated as the changes in Δ*H°*, Δ*S°*, Δ*G°*_37_ and Δ*G°*_60_. The thermodynamic parameters of reference DNA/DNA duplexes used here were predicted by using the hybrid-2s.pl program in the UNAfold software package [24] under the salt condition used in the experiments. For single base pair mismatches, there are positional stability differences for the mismatches [25–27], which are not considered by the UNAfold software package. Therefore we adjusted the UNAfold-derived DNA/DNA thermodynamic parameters by using observed values. For a certain DNA/DNA pair, we used ECHO/DNA pairs of the same base composition, in which the labelled bases locate more than three-base away from the mismatch

$$\Delta H_{\left(\text{DNA}/\text{DNA}\right)}^{o}\;=\Delta H_{pred\left(\text{DNA}/\text{DNA}\right)}+\frac{1}{N}\underset{d>3}{\sum }\left\{\Delta H_{obs\left(\text{ECHO}/\text{DNA}\right)}^{o}-\Delta H_{pred\left(\text{ECHO}/\text{DNA}\right)}^{o}\right\}$$

where *pred* denotes the predicted value by UNAfold and previously reported ECHO/DNA parameters, *obs* denotes the observed value obtained by this study, *d* denotes the distance between the labelled base and mismatch base pair, and *N* denotes the number of ECHO/DNA pairs.

Then we modeled the incremental thermodynamic parameters, ΔΔ*H°*, as the effect of three nucleotides including T^E^ (NT^E^N), which can be decomposed as the combination of two nearest neighbors (NT^E^ and T^E^N)

$$\Delta \Delta H^{o}≅\Delta \Delta H_{{\text{NT}}^{\text{E}}\text{N}}^{o}=\Delta \Delta H_{{\text{NT}}^{\text{E}}}^{o}+\Delta \Delta H_{{\text{T}}^{\text{E}}\text{N}}^{o}$$

The nearest neighbor incremental parameters (ΔΔ*H°*_NT_^E^ and ΔΔ*H°*_T_^E^_N_ above) for a single base pair mismatch next to the labelled base were obtained by using 24 ECHO/DNA duplexes covering all possible single-base mismatches next to the labelled base. Multiple linear regression analysis using singular value decomposition described previously [22] was applied separately to the sets of ΔΔ*H°*, ΔΔ*S°*, ΔΔ*G°*_**37**_, and ΔΔ*G°*_**60**_ to yield the incremental nearest neighbor thermodynamic parameters of the dinucleotides: one parameter for 5’ side mismatch NT^E^/AN (AT^E^/AV, CT^E^/AH, GT^E^/AD, TT^E^/AB) and one parameter for 3’ side mismatch T^E^N/NA (T^E^A/VA, T^E^C/HA, T^E^G/DA, T^E^T/BA).

### Statistics

For comparing two groups the unpaired two-tailed Student’s T test was used. All tests were performed with R version 2.12 [23].

### Development of Edesign software

Edesign is based on the Primer3 software (Primer3 core C program version 2.3.4 and Primer3 web interface version 3.0.0), for which the source code was downloaded at http://sourceforge.net/projects/primer3/files/primer3/, and for the web interface at http://sourceforge.net/projects/primer3/files/primer3-web/.

The Edesign core program has a similar structure as the Primer3 core program (each library name has extension '_Z' at the end of the original program library). The schematic structure of Edesign’s core program is shown in S1A Fig. In short, the Edesign core program consists of primer3_boulder_main_Z: the root library that controls of the core program, read_boulder_Z: library for reading input parameters and files, format_output_Z and print_boulder_Z: libraries for writing outputs, oligotm_Z: library that calculates the *T*_M_ of oligonucleotides, dpal_Z: library that calculates old-style alignments, thal_Z: library that calculates thermodynamic alignments, and the library libprimer3_Z, which provides Edesign’s central design capabilities. When called to design, libprimer3_Z’s central function, choose_primers(), performs an exhaustive search for the 'best' primer and internal probe combinations, given the target sequence and other input parameters (details of the design workflow is summarized in S1B Fig). The notion of ‘best’ combinations is operationally defined as minimizing a penalty score. Edesign web interface consists of three main components, index.htm: the input form, web_help.htm: the help page and results.cgi: CGI program that runs Edesign core program.

The core program codes were modified by adding new codes written in C for enhancing internal probe design, enabling genotyping design, and treating modified oligonucleotides. The web interface was modified by new codes written in perl, html and JavaScript. These added parameter fields and procedures relate to new functions that are summarized in S1 Table. In brief, the internal probe search algorithm was totally rebuilt to conduct probe designs on both DNA strands and to evaluate internal probes with the same priority as primer pairs (implemented in read_boulder_Z and libprimer3_Z). In the internal probe evaluation procedure, a sequence specificity check and primer-probe complementarity check were added (implemented in libprimer3_Z). A genotyping design mode was established to have the option to design further internal probes for genotyping assays, where Edesign estimates *T*_M_ values of internal probes for a user-defined genetic variant, and evaluates it for better discrimination between the wild-type sequence and the variant (implemented in primer3_boulder_main_Z, read_boulder_Z and libprimer3_Z. The *T*_M_ for the variant is displayed through format_output_Z). For working with a modified oligonucleotide, read_boulder_Z, format_output_Z, print_boulder_Z and libprimer3_Z were extended to treat the additional modified-nucleotide (code ‘Z’). Subprograms oligotm_Z, dpal_Z and thal_Z were also extended for ‘Z’, and each of them can work as a single independent tool as in Primer3. Thermodynamic parameter files (in primer3_config_Z directory) for thal_Z and thermodynamic parameters coded in oligotm_Z were extended by the nearest neighbor parameters of the modified nucleotide ‘Z’ obtained in the previous study [22] and this study (thiazole orange double-labelled thymidine). The Excel tool that facilitates the update of those thermodynamic parameters was developed using Visual Basic for Applications, and was used to update the thermodynamic parameters. ECHOs have positional preferences for the labelled nucleotide, and self-folding features have to be considered during probe design. Those characteristics of ECHO were implemented as design functions (implemented in read_boulder_Z and libprimer3_Z), and the default input parameters for the functions were set based on the experimental results (set in input.htm). The new source code for Edesign can be downloaded at https://github.com/yasumasak/edesign/ under a GNU General Public License version 2.0 (GPLv2). The software is available for UNIX, LINUX and Mac OS X platforms using an ANSI C compiler.

### Eprobe mediated qPCR and melting curve experiments

Eprobe mediated qPCR and melting curve experiments were performed as described in [17]. Details on the primers, probes and PCR settings are provided in the S7 and S8 Figs. The slopes in the standard curves and the PCR efficiencies were calculated as described in [17].

## Results and Discussion

### New features for designing internal probes

**Fig 2:**
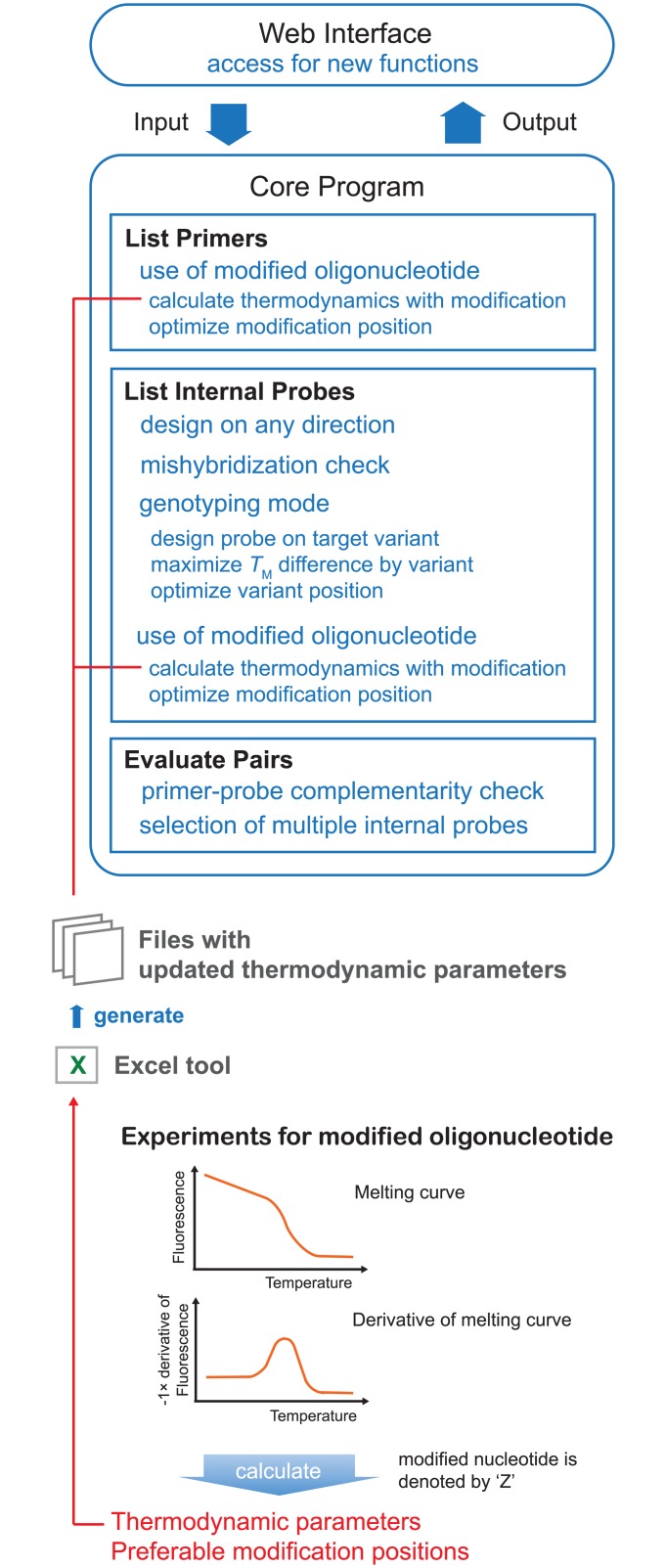
Schematic work flow of Edesign. New functions for “Web Interface”and “Core Program” are shown. Thermodynamic parameters for modified oligonucleotides can be updated through the Excel tool after obtaining a set of nearest neighbor parameters.

While very good programs are available for the design of PCR primers, we saw a need for a better tool for selecting internal probes for qPCR experiments. Furthermore, the tool should be suitable for developing internal probes for genotypic assays that are often conducted by melting curve analysis of the PCR product. Our new design tool, Edesign, was developed using the established primer design software Primer3 (Primer3 core C program version 2.3.4 and Primer3 web interface version 3.0.0). Primer3 is widely used and we selected the program because it offers detailed settings for use in various PCR applications. We modified Primer3 as shown in Fig 2 (refer to S1 Table for a complete list of new functions in Edesign) to improve the selection of internal probes (refer to S1 Fig for a description of schematic structure of the Edesign core program). Primer3 focuses on primer selection where the final output is ranked by scores of primer pairs while one internal probe is additionally picked for each primer pair on the forward strand only. This had been revised in Edesign, which allows both orientations for binding of an internal probe to either strand. Moreover, in Edesign the internal-probe selection function is checking for binding to similar sequences within the target sequence to avoid mis-hybridization, and looks for possible complementary sequences between the primers and the probe. The final primers-internal probe sets in Edesign are selected based on total scores for each primers-internal probe set. Edesign provides the optimal primer and internal probe sequences based on a target sequence defined by the user. The user has the option to specify within the target sequence regions that should be specifically excluded or included into the areas where the selected primers and internal probe will bind. This option is important when working for example with viral sequences that can have high mutation rates in certain regions.

An additional function was added to Primer3 in Edesign for selecting probes for detection of DNA variations in genotyping assays (“Genotyping Mode”). Using this function, the user can obtain an internal probe over a user-specified target genetic variation within a target sequence. Edesign can define probes for the detection of individual SNPs, point mutations, insertions and deletions, or any nucleotide variation surrounded by sequences to which the internal probe can specifically bind. The program calculates the melting temperature (*T*_M_) of an internal probe for binding to the wild-type sequence and the sequence carrying the genetic variation to estimate the difference between the *T*_M_ values. The *T*_M_ values are calculated using the unified nearest-neighbor thermodynamic parameters [28] that considers mismatches in dinucleotide combinations and treats stabile mismatches at the terminal ends of the sequence differently from destabilizing mismatches within the oligonucleotide. Aside from the nearest-neighbor model there are positional stability differences for the mismatches within oligonucleotides [25–27], a mismatch in the middle of the probe tends to have larger destabilizing effect than mismatches at the ends. However, Edesign does not prioritize the position of the mismatch within the probe. We omitted this function, because such positional stability effects for the mismatches do not only depend on the position of the mismatch within the probe but also on the base composition around the mismatch [25–27]. Instead, Edsign provides a function to specify the length of the terminal regions of the probe where no mismatches will be placed. This offers the user an option to locate the position of the mismatch rather in the center of the probe for achieving a higher *T*_M_ difference. At the end of the design process the internal probe with higher *T*_M_ difference will be selected in Edesign under the default settings for optimal discrimination between the wild-type and the variant, where penalty scores consider *T*_M_ values, complementarity, and mis-hybridization weight matrixes. Edesign is searching for a probe with a high *T*_M_ difference, and therefore will give a clear priority to shorter oligonucleotides having at the same time a low *T*_M_ value. Note that during the amplification reactions, the PCR instruments commonly measures the fluorescent signal only at one temperature. To avoid *T*_M_ values that are lower than the detection temperature set on the PCR instrument, Edesign allows to set a limit for the *T*_M_ values of the variant as well as the wild-type sequence. While short probes with a larger *T*_M_ difference are suitable for working with Eprobes, these short oligonucleotides may not work as TaqMan probes that require *T*_M_ values in the range of the temperature of the extension reaction. Hence for the design of TaqMan probes the *T*_M_ value of the probe should be set higher in Edesign than given under the default setting.

**Fig 3:**
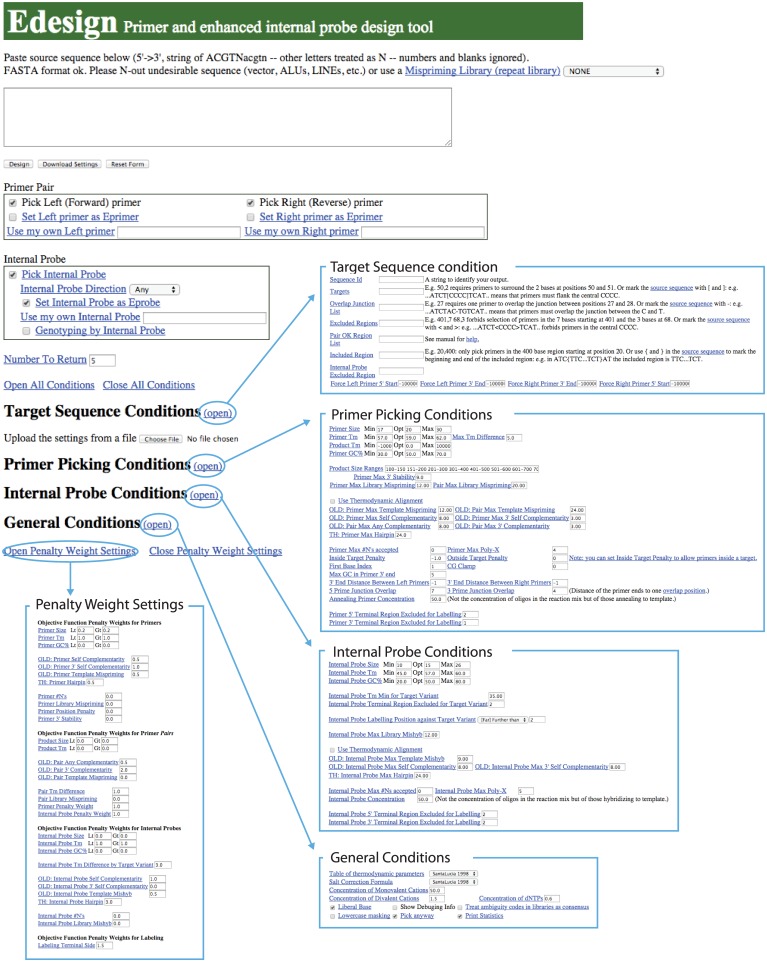
Web interface of Edesign. Design parameter fields are hidden and are only shown when a link to the corresponding category is checked. Changed parameter settings can be saved on a local disk and the saved setting can be reloaded for later use.

**Fig 4:**
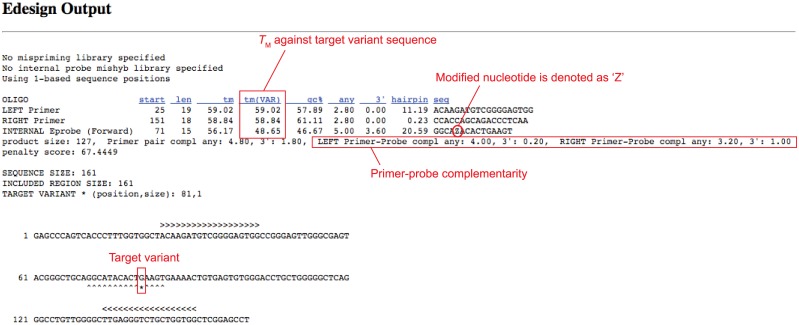
Example of the output page of Edesign using the genotyping mode.

The new web user interface for Edesign is shown in Fig 3. The user can define the design parameters consistently with the parameters used in Primer3, where categories were divided into “Target Sequence Conditions”, “Primer Picking Conditions”, “Internal Probe Conditions”, and “General Conditions” to improve the visibility of those parameters. The user can change on the web interface each of the design parameters and save the settings for reuse on a local disk. After running the program using the user-defined settings, Edesign will provide the output in a page that is similar to the Primer3 result page (Fig 4). When applying the new genotyping mode, the output page will additionally provide the estimated *T*_M_ value of the internal probe for binding to the target variant and the wild-type sequence to facilitate evaluation of the variant-discrimination ability.

Edesign uses the unified thermodynamic parameters for DNA [28], the standard parameters in Primer3, for designing regular DNA oligonucleotides. Therefore Edesign can be used for the selection of any type of internal probe that does not change the DNA binding affinity in the hybridization step. This includes the most commonly used regular TaqMan probes, which can be designed in Edesign. However, the thermodynamic parameters used for standard DNA oligonucleotides are not sufficient for designing internal probes with altered DNA binding affinities such as Eprobes, where the dye moieties interact with the DNA strands. Therefore we developed an Excel Tool for Edesign that allows the user to create the thermodynamic parameters for using other types of binding molecules in Edesign (Fig 2). As an example for using the Excel Tool, we studied the thermodynamic properties of thiazole orange-labelled ECHOs to acquire the data needed for optimizing Edesign for Eprobe mediated qPCR and genotyping experiments.

### Positioning of labelled nucleotide within Eprobes

**Fig 5:**
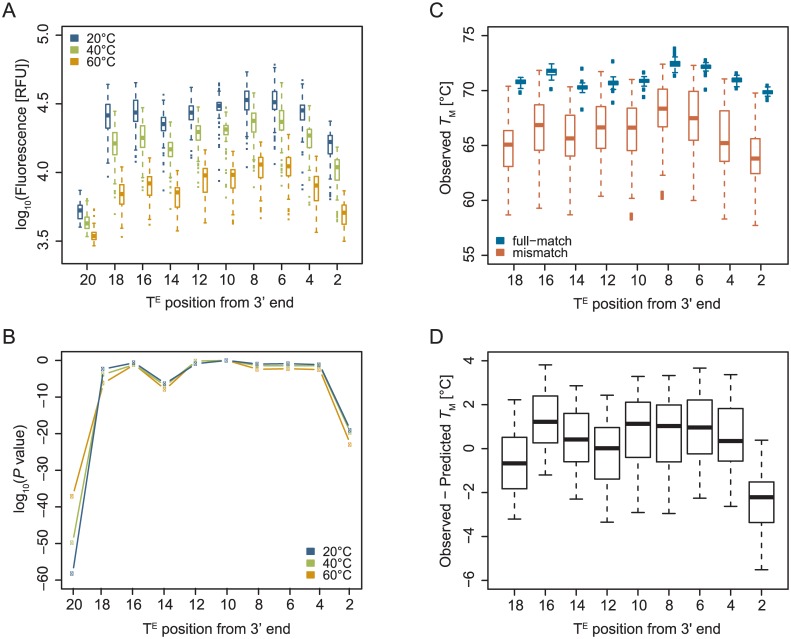
Positional effect of thiazole-orange labelled nucleotide within ECHO on (A) fluorescence intensity (at 20, 40 and 60°C) and its *P* value (B) by comparing with ECHO labelled at the center (position 10) (Student’s T test, two-tailed), (C) *T*_M_ obtained from melting curve analysis (boxplots separated by with or without mismatch), and (D) difference between the predicted and observed *T*_M_ values. In graph A, each boxplot summarizes fluorescent melting curve experiments of one ECHO with 61 complementary oligonucleotides (one full-match and 60 single-base mismatch sequences), using mean values among replicates of each ECHO/DNA pair. In graph C, each boxplot summarizes obtained *T*_M_ values of one ECHO with one full-match or 60 single-base mismatch sequences, using all replicates. In graph D, each boxplot summarizes differences between predicted and obtained *T*_M_ values of one ECHO with single-base mismatch sequences, whose labelled nucleotide located more than two nucleotides away from a mismatch, using mean values among replicates of each ECHO/DNA pair.

In our previous analysis of thiazole orange-labelled ECHOs, we had placed the labelled thymidine in the center of the oligonucleotide to obtain the nearest neighbor thermodynamic parameters for calculating the *T*_M_ values of ECHOs [22]. The base composition around the labelled thymidine had no influence on the fluorescence signal intensity [29], however, whether a labelled nucleotide has the same effect on the *T*_M_ and fluorescent signal in any position of an oligonucleotide remained unknown. Therefore we randomly selected a 20-mer sequence with a thymidine in every second position, and synthesized 10 different ECHOs having the same sequence but the label at positions 2, 4, 6, 8, 10, 12, 14, 16, 18 or 20 counted from 3’ end of the oligonucleotide to look for positioning effects of the labelled nucleotide. Those ECHOs were analyzed in fluorescent melting curve experiments with 61 complementary oligonucleotides (one full-match and 60 single-base mismatch sequences). The fluorescent signals obtained at 20, 40 and 60°C were plotted against the position of the labelled nucleotide (Fig 5A) to compare them while the double strand is fully stable (20°C), at an intermediate temperature (40°C), and at temperatures used in PCR reactions (60°C). The plot revealed clearly reduced intensities for labels located at the end of the oligonucleotides (positions 2 and 20) that were statistically significantly different when compared with a label located at the center (position 10) (Fig 5B). Since the signal for a label in last position at 5’ end (position 20) was by far the lowest, this position is not suitable for designing ECHOs or Eprobes. We further analyzed the peak height of negative first derivative of the melting curves (S2A Fig), where the fluorescence intensity mostly correlated with the height of the negative first derivative (S2B Fig). To confirm the decrease of fluorescence signals for labels at terminal positions, two additional ECHOs of different length (15-mer and 25-mer) were prepared with three thymidines at each end (S3 Fig). Compared to a label within the center of each ECHO, reduced fluorescent signals were observed for labels in any of the last 3 positions at the 5’ or 3’ end, where the strongest decrease in the fluorescence was observed again in the end positions of the oligonucleotides. The positioning effect was already much lower for labels in second or third position from the end, indicating that positions from the third position onwards are suitable for positioning the labelled nucleotide (default setting in Edesign). From the melting curves the thermodynamic parameters of duplex stability, Δ*H°* and Δ*S°* can be obtained to give the *T*_M_ for each duplex (Fig 5C). Using the previously reported nearest neighbor parameters [22] and ECHO/DNA pairs whose labelled nucleotide located more than two nucleotides away from a mismatch, we further predicted the *T*_M_ for each ECHO/DNA pair and compared them with the *T*_M_ values from the observed melting curves; the *T*_M_ value for the label in position 20 was excluded because of the weak fluorescence signal. As shown in Fig 5D, we found a good agreement between the predicted and observed *T*_M_ values over most parts of the oligonucleotides (positions from 4 to 18, observed–predicted *T*_M_ = 0.52 ± 1.6°C), where again the most terminal position was not favored (position 2, observed–predicted *T*_M_ = -2.4 ± 1.5°C, *P* < 10^−18^ when compared with positions from 4 to 18).

The foregoing experiments showed that the labelled thymidine should not be placed in the last two positions at the 5’ or 3’ end of the oligonucleotide. The benzothiazole and quinolinium rings of the thiazole orange moieties need to be locked in a plane position within the double-stranded DNA for obtaining strong fluorescence [30,31]. Therefore we assume that thiazole orange moieties attached to nucleotides in the first or second position from the ends cannot properly intercalate into the double strand. Incomplete intercalation of one or even both dye moieties could lead to the low fluorescence intensity and the reduced *T*_M_ values observed in our experiments.

### Positioning of mismatch within Eprobes

**Fig 6:**
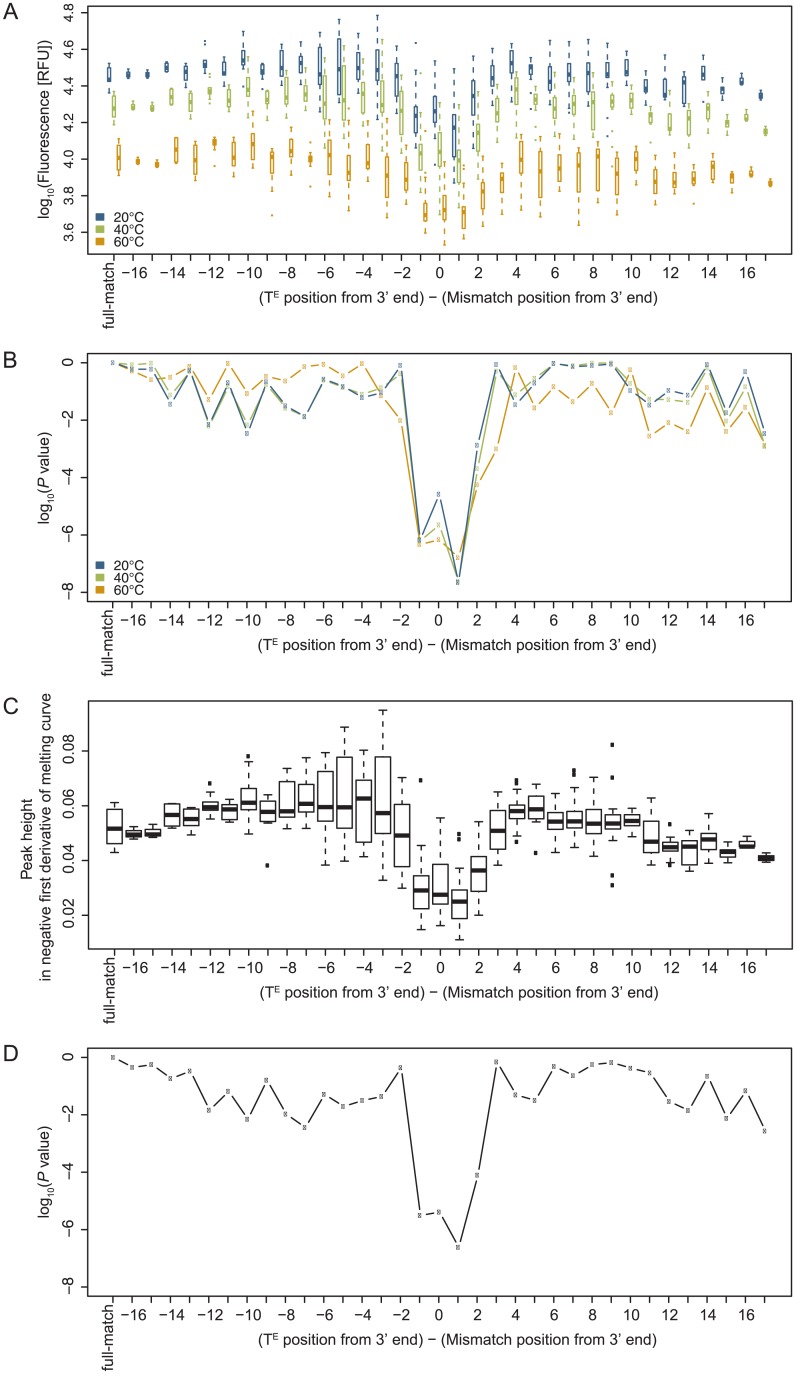
Effect of mismatched base pair positions relative to the thiazole-orange labelled nucleotide on (A) fluorescence intensity (at 20, 40 and 60°C) and its *P* value (B) by comparison to full-match ECHO/DNA hybrids (Student’s T test, two-tailed), and (C) peak height in negative first derivative of melting curves and its *P* value (D) by comparison to full-match ECHO/DNA hybrids (Student’s T test, two-tailed). Each boxplot and point in (A-D) summarizes ECHO/DNA pairs with different ECHOs having the same distance from the labelled nucleotide to a mismatch, using mean values among replicates of each ECHO/DNA pair.

Edesign allows designing hybridization probes dedicated for the detection of genetic variations. Therefore we analyzed positioning effects for mismatches in Eprobes to better understand where to place a mutation in relationship to the labelled nucleotide. We used the same set of ECHOs having a thymidine in every second position to test all possible combinations for the 8 labelling positions (excluding positions 2 and 20) versus mismatches in each position of the complementary oligonucleotide. In total 60 different antisense oligonucleotides and 480 ECHO/DNA combinations were applied for conducting melting curve experiments under the same conditions described above. For each of the 8 ECHOs we obtained the fluorescence intensity for a mismatch in each position in the complementary oligonucleotide and compared the values to the signal obtained with the fully-matching oligonucleotide (Fig 6A and 6B). We further performed the same analysis for the peak height of the negative first derivative of the melting curves (Fig 6C and 6D), which showed the same tendency as observed for the fluorescence intensity. The results show that the fluorescence intensity is clearly reduced for mismatches located in position -1, 0, +1 and moderately reduced in position +2 from the labelled nucleotide (5’ to 3’ direction). This observation was further confirmed by the analysis of a 15-mer as further outlined in S4 Fig. Most likely a nearby mismatch offers more freedom for the benzothiazole and quinolinium rings in thiazole orange to rotate instead of taking up a fixed position in the plane between the bases. This rotation allows non-radioactive relaxation of the excitation energy [30,31] leading to a reduction of the fluorescent signal. The pattern seen for the reduced signal could be asymmetric, where the two dye moieties may intercalate between positions -1 and 0, or +1 and +2 from the labelled nucleotide in position 0. This observation confirms previous results on putative intercalation positions using locked nucleic acid (LNA) incorporated into ECHOs [32]. The observed signal distribution in our experiments has to be taken into account when designing an optimal Eprobe for genotyping assays. In standard genotyping experiments a strong fluorescent signal is desirable to conduct melting curve analysis and to determine the peak position and *T*_M_ value. Therefore it is preferable to place the labelled nucleotide far away from the mismatch position; under default settings Edesign will place the labelled nucleotide at least three or more nucleotides away from the mutated position. However, it is also possible to design alternative genotyping assays, where the fluorescent signal is only observed for the wild-type DNA, while the signal will be lost or at least reduced when the probe hybridizes to the mutated sequence. For such assays Edesign offers settings to design probes where the labelled nucleotide is close to the mismatch (default value in Edesign set at within one nucleotide of the mutated position). The users can select either option for designing a probe, where the default settings can be adjusted in the Edesign interface.

In the previous experiments we included every possible mismatch in each position of the oligonucleotide, which allowed us to determine the nearest neighbor parameters for mismatched bases next to the labelling position (T^E^). Two sets of nearest neighbor parameters were calculated by multiple linear regression analysis using singular value decomposition for each mismatch at the 3’ side of the labelled position (5’-T^E^N-3’) and each mismatch at 5’ side (5’-NT^E^-3’) with N indicating the position of the mismatch (Table 1, further details are provided in S2 Table). The incremental effects on enthalpy and entropy varied but the incremental effects on the free energy were in a constant range and about equal to those for fully matching base pairs. The data indicate that one of the two dye moieties can intercalate in the double-stranded DNA into a position adjacent to a mismatched base pair, and has thus a stabilizing effect on the duplex. The nearest neighbor parameters obtained in these experiments were incorporated in Edesign for calculating *T*_M_ values for any ECHO/DNA duplex including those ECHO/DNA duplexes that have a labelled nucleotide next to a mismatched base pair.

**Table 1:** Thermodynamic parameters for mismatch next to labelled thymidine (one parameter for 5’ side mismatch NT^E^/AN (AT^E^/AV, CT^E^/AH, GT^E^/AD, TT^E^/AB), and one parameter for 3’ side mismatch T^E^N/NA (T^E^A/VA, T^E^C/HA, T^E^G/DA, T^E^T/BA)).

nearest neighbor	ΔΔ*H°*	ΔΔ*S°*	ΔΔ*G°*_*37*_	ΔΔ*G°*_60_
(5' to 3' / 5' to 3')	[kcal mol^-1^]	[cal mol^-1^ K^-1^]	[kcal mol^-1^]	[kcal mol^-1^]
NT^E^/AN	-3.6 ± 1.4	-4.4 ± 2.4	-2.3 ± 0.4	-2.3 ± 0.3
T^E^N/NA	-2.9 ± 1.5	-3.2 ± 2.6	-2.0 ± 0.5	-2.2 ± 0.3

### Effect of self-folding within Eprobes

**Fig 7:**
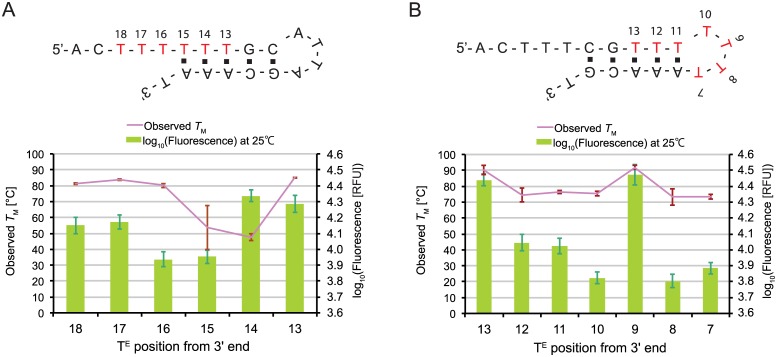
Effect of self-folding on *T*_M_ and fluorescence intensity at 25°C. (A) Effects of the labelling positions in the stem and outside of the stem-loop region. (B) Effects of the labelling positions within the stem and internal loop region.

Self-folding of ECHOs and Eprobes can cause high background signals when dye moieties start to interact with regions forming a double-stranded stem structure. To investigate the background signal from different positions of the labelled nucleotide within and around a stem structure, we designed two basic sequences (SELF_A: 5’-ACTTTTTTGCATTAGCAAAT-3’, SELF_B: 5’-ACTTTCGTTTTTTTAAACGT-3’) that can form stable stem loop structures and have thymidines within the single-stranded overhang, the stem and the loop. For each sequence several labelled oligonucleotides were prepared with thiazole orange-labelled thymidines in different positions (Fig 7). All ECHOs were analyzed by melting curve analysis and we measured the fluorescent signal and *T*_M_ values for labelling positions within the single-stranded region and the stem structure (Fig 7A), and within the stem structure and the single-stranded loop (Fig 7B). In line with our previous data on the positioning effect of the label, we observed again a stronger signal for labelling positions in the center of the stem structure as compared to labelling the last two positions within the stem. Unexpectedly, however, a high fluorescence and *T*_M_ value were also observed with oligonucleotide SELF_A for labelling positions 18 and 17 within the single-stranded overhang (Fig 7A), where the signals were nearly in the same range as for a labelling position in the center of the stem structure (positions 13). With oligonucleotide SELF_B, we further observed a strong signal for a labelling position within the loop structure (position 9) that showed an equal *T*_M_ value as compared to position 13 within the double-stranded stem (*P* > 0.1, Fig 7B). To confirm the observation that dye moieties placed outside of the stem region can emit fluorescence, we prepared a different pair of partially-complementary oligonucleotides, where the labelled nucleotide was placed again in the single-stranded overhang (S5 Fig). Since we also observed with this pair a fluorescence emission, we speculate that dye moieties from outside may interact with the distal stem region forming some high-order structure. We observed only a weak stabilizing effect on the double strand for the studied pair, but we cannot rule out by the analysis of only one oligonucleotide pair that such an interaction could also significantly change the *T*_M_ of other hybrids. Thus these results confirm that strong self-folding of Eprobes is unfavorable, where even dye moieties located outside of a stem region could possibly interfere with the DNA structure and cause background signals. Therefore we addressed this point in Edesign.

### Prediction of self-folding structures

As outlined above, compared to standard DNA probes, Eprobes have specific thermodynamic features that have to be taken into account during assay design. While thiazole orange as an intercalating dye increases the binding affinity, there are restrictions on positioning of the label, and any self-folding or partially complementary regions have to be avoided. Hence we wrote a self-folding prediction program for ECHOs and Eprobes by combining the obtained thermodynamic parameters for ECHO/DNA duplex formation with the thermodynamic alignment program thal in Primer3 [8]. The thal program can predict duplex and self-folding thermodynamic stability, considering full-matches, mismatches, dangles, bulges, internal loops and hairpin loops, and is used for calculating *T*_M_ values both for desired and undesired positions within a target sequence. The full-match and mismatch nearest neighbor parameters for ECHO/DNA duplexes were updated by combining our new data with those from our previous studies [22]. The new parameter files were generated by using the Excel tool and linked to the updated version of thal (thal_Z) in Edesign to calculate the *T*_M_ values for self-folding structures and the hybridization of the Eprobe to its target sequence. For evaluation of the program, we designed several ECHOs, which can form secondary structures, and measured their melting curves. The experimentally obtained *T*_M_ values for their double-stranded regions were compared with the predicted *T*_M_ using our new prediction method (S3 Table). The differences between the observed and predicted *T*_M_ values were in the range of -1.9 ± 12.3°C. The prediction performed overall well, but some sequences showed large differences from the prediction leading to a high error margin. It seems this high error is largely caused by an unclear behavior of the dye moieties when located outside of the stem region. Therefore the dangle, bulge, internal and hairpin loop parameters within the program could be updated after more data become available. However, Edesign is set to avoid stable secondary structures for all oligonucleotides under the present default settings putting a high penalty weight on the probe hairpin *T*_M_ value. With a hairpin *T*_M_ value set lower than the estimated standard deviation, the accuracy of the present *T*_M_ prediction is sufficient for Eprobe designs. In addition, the user can change the default setting in Edesign when designing very short probes, where the maximum *T*_M_ for the hairpin should be around 20°C lower than the minimum *T*_M_ of the probe.

### Eprobe-specific features implemented in Edesign

Using the thermodynamic data from our experiments and the Excel Tool, we updated Edesign for the use of thiazole orange-labelled Eprobes, and added default settings for using the optimal parameters. S1 Table summarizes the Eprobe-specific features that were implemented into Edesign. These features allow users to specify Eprobe design options where the program will examine and prioritize based on a penalty score all the available dye-labelling positions. Examined labelling positions are by default more than two nucleotides from the 5’ or 3’ end of the oligonucleotide, although the setting can be changed by the user. To avoid high background fluorescence of Eprobes, Edesign further calculates *T*_M_ values for hairpin structures within oligonucleotides, and removes sequences having higher *T*_M_ values than the specified threshold (default threshold value in Edesign is 24°C). The same thermodynamic parameters are used to search for hairpin structures and to calculate *T*_M_ values for ECHO/DNA or Eprobe/DNA hybrids when binding to a target sequence or a complementary oligonucleotide.

The user has the choice to design primers/probe sets using standard DNA oligonucleotides as a probe or Eprobes including special options to design probes for genotyping experiments. When working with Eprobes, however, the user can in addition specify the labelling position within the Eprobe either near the sequence variance to lower the fluorescent signal by the mismatch, or far from the target variance to obtain an optimal signal as outlined above. S6 Fig provides more information on how Eprobe-mediated signals change depending on the position of the mismatch, and how the results should be interpreted. For standard qPCR and genotyping experiments we recommend to use the default setting, where the mismatch is placed far away from the labelling position. Users can further enter their own primer/probe sequences into Edesign, where Edesign will select the optimal labelling position within the user-defined Eprobe sequence; labelling positions can also be designed for a primer (so-called Eprimer). This function could be useful when published primer pairs or probes should be used in an experiment.

**Fig 8:**
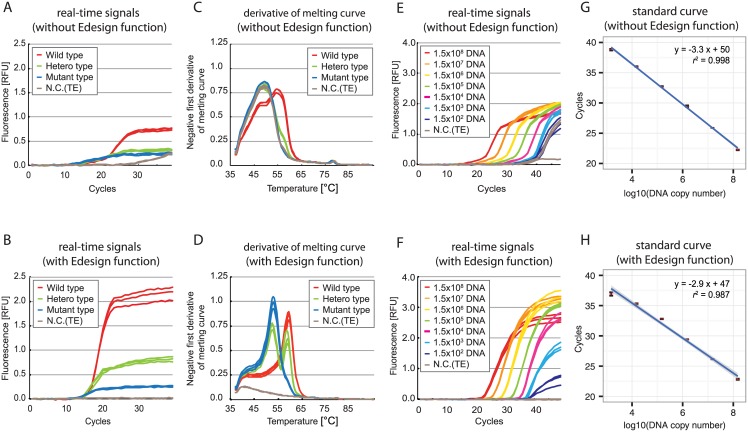
Evaluation of Eprobes and primers designed by Edesign. (A-D) Evaluation for mutation detection using pandemic 2009 H1N1 influenza virus genome segment 6 (neuraminidase) H275Y mutation; (E-H) Evaluation for low copy DNA detection using influenza B virus genome segment 7 (matrix protein) as a template. Upper panels (A, C, E, G) show results obtained by using Eprobes and primers designed by Primer3 with updated tables for *T*_M_ calculation of ECHOs. Lower panels (B, D, F, H) show results obtained by using Eprobes and primers designed by Edesign. Details on primers and Eprobes used are provided in S7 and S8 Figs.

The new Eprobe-specific features in Edesign were tested by designing primers/Eprobe sets for two complicated target sequences using Edesign under the default setting and Primer3 version 2.3.4 [7,8] with the previously reported ECHO thermodynamic parameters [22] not including the new data and features developed during this study; details on the designed primers and probes are given in S7 and S8 Figs. PCR conditions used directly the parameters obtained from the design tools, and no further efforts were made to better optimize the PCR reaction conditions. The qPCR and melting curve experiments were performed under the standard conditions we had established for Eprobe-mediated PCR [17]. Fig 8 shows the real-time PCR results for point mutation detection in the pandemic 2009 H1N1 influenza virus genome segment 6 (neuraminidase, H275Y mutation) sequence (Fig 8A, 8B, 8C and 8D) and for low copy number detection of the influenza B virus genome segment 7 (matrix protein) sequence (Fig 8E, 8F, 8G and 8H). For both targets primers/Eprobe sets designed by Edesign showed that the sensitivity was improved with a lower background signal as compared to the reference primer and probe set (compare Fig 8B and 8F to 8A and 8E). For the H275Y mutation in the neuraminidase gene we further conducted a melting curve analysis on the PCR products for mutation detection, where the Eprobe designed by Edesign provided a much higher resolution and better separation of the peaks (compare Fig 8D to 8C). The results of both experiments indicate that Edesign in combination with our new thermodynamic parameters under default conditions offers large improvements for Eprobe designs over Primer3 combined with our previous data. These conditions are suitable for using Edesign for conducting genotyping and qPCR studies as shown here for the design and analysis of viral target sequences where the regions available for primer and probe design were restricted by frequent genetic variations.

### Other considerations using Edesign

Edesign provides additional features that can be useful for working with TaqMan probes rather than Eprobes, or for working with PCR primers having a mismatch at the 3’ end. For designing allele-specific primer sets, primers are used where the last nucleotide at the 3 ‘end falls onto a sequence variation between both alleles. Because of the mismatch in the last position of the primer, the primer extension reaction can only occur for the sequence having a fully matching sequence in the last position. To assist the user to design allele-specific primers, Edesign has a function to fix the position of the forward and/or reverse primers. These positions can be selected in such a way that the 3’ end of a primer falls onto a sequence variation, thus allowing for allele-discrimination by the selected primer set. However, since Edesign will provide the fully matching primer sequences in the output file, the user will have to manually select the adequate mismatch for the 3’ end position of the primer(s).

Regular DNA oligonucleotides do not show a stabilizing effect on the DNA binding as seen for Eprobes, and therefore may require other temperature settings for probe design. Therefore *T*_M_ values and length settings for an internal probe can be changed in Edesign as needed to obtain optimal probe sequences. For TaqMan probes for example, the required *T*_M_ values must be in the range of the temperature for the extension reaction. Hence for the design of TaqMan probes *T*_M_ and length values of the probe should be set higher and longer in Edesign than given under the default setting. Otherwise, the TaqMan probe may not be degraded during the elongation step and only a weak or even no signal at all will be observed during the amplification process. Edesign can also be used for applying regular DNA oligonucleotides or TaqMan probes in genotyping experiments. The user can restrict the regions where the mismatch will be placed within the probe. The destabilizing effect of a mismatch is depending on the base pair type with a clear trend for the order of decreasing stability: G-C > A-T > G·G > G·T ≥ G· A > T·T ≥ A·A > T·C ≥ A· C ≥ C·C [33]. Guanine is the most promiscuous base, since it forms the strongest base pair and the strongest mismatches. On the other hand, cytosine is the most discriminating base, as it forms the strongest matching pair and the three weakest mismatches. Edesign will select that strand for the probe, which has the higher discrimination ability between the full match and mismatch considering also other factors such as sequence complementarity and the *T*_M_ value.

### User defined extension options in Edesign

With a focus on the use of Eprobes in PCR experiments, we have extended the functions in Edesign for providing thiazole orange-labelled Eprobe designs. However, it is envisioned that other dyes used in Eprobes will have other thermodynamic features that will have to be individually determined. Therefore we prepared a framework to implement such features also for other modified oligonucleotides into Edesign (Fig 2). The Excel tool update_thermodynamic_parameters.xlsx within the Edesign package can generate the thermodynamic parameters and program files needed for updating Edesign. The required input for the Excel tool is a set of the incremental nearest neighbor parameters, ΔΔ*H°* and ΔΔ*S°* values, which can be obtained from a set of melting curve experiments as described here and in other studies [22,34]. The modification-oriented settings can then be changed within the parameter fields of the interface. The default settings for those parameters can be changed by modifying the html file index.htm for the interface. Thus a customized Edesign core program and interface for other modified nucleic acids or nucleic acid analogues can easily be made after obtaining the necessary thermodynamic parameters and possibly additional data on positioning effects. This function will be used in the future to add more dyes for Eprobe labelling and other possible probe designs to Edesign.

## Conclusion

Edesign is a new tool for the design of primer sets and internal probes for conducting qPCR and genotyping experiments. Particular efforts were made to implement also the design of thiazole orange-labelled Eprobes. These Eprobes offer great benefits for genotyping assays because of their higher binding affinity as compared to standard DNA oligonucleotides. We provided with this study an example on how new Eprobes having other dyes or internal probes using an entirely different chemistry can be characterized for better probe designs. With the Excel Tool within the Edesign package we provide a convenient tool to incorporate new probe designs into our primer and probe design tool. Tests on primers and Eprobes designed by Edesign in PCR experiments confirmed the improvements of Edesign over the original version of Primer3, which cannot calculate *T*_M_ values for Eprobes and had other limitations on the design and evaluation of internal probes. Edesign has already been used in a number of projects for the successful establishment of genotyping assays for medical studies (YK unpublished data). Most of those studies required the analysis of multiple SNPs or genetic variations emphasizing the importance of reliable computational tools for assays design.

Edesign is freely available at https://github.com/yasumasak/edesign/ for downloading the source code, which allows running Edesign as a stand-alone software or as a web application. In addition, the Edesign web interface can be publicly accessed at http://www.dnaform.com/edesign2/ for free usage without limitations. With the download, a README file is provided that contains instructions on how to install and use the software (https://github.com/yasumasak/edesign/blob/master/README). In addition, we are providing a help document on the website to assist users to make best use of Edesign (http://www.dnaform.com/edesign2/web_help.htm). The help document advices on the use of Edesign and the parameters set in the tool are described.

The implementation of the Excel Tool within Edesign offers options to update and further customize the program for the use of other probe designs. This may include the use of modified DNA backbones, intercalators added to a probe, or new dyes made available for Eprobes. With the new features and improvements over Primer3, we hope Edesign will be useful for the community to setup their PCR and genotyping experiments.

## Supporting Information

S1 Data Melting curve data. (TXT)

S1 Fig Schematic structure of Edesign core program. (PDF)

S2 Fig Summary of peak heights of negative first derivative of melting curves. (PDF)

S3 Fig Fluorescence signals of 15-mer, 20-mer and 25-mer ECHOs. (PDF)

S4 Fig Effect of mismatched base pair positions relative to the thiazole-orange labelled nucleotide in 15-mer ECHO/DNA hybrids. (PDF)

S5 Fig Fluorescent signal of a dye moiety locating outside of duplex region. (PDF)

S6 Fig Interpretation of Eprobe mediated PCR and melting cure experiments (PDF)

S7 Fig Designed Eprobes and primers for evaluation in mutation detection. (PDF)

S8 Fig Designed Eprobes and primers for evaluation in low copy number detection. (PDF)

S1 Table New Functions in Edesign. (PDF)

S2 Table Thermodynamic parameters used for nearest neighbor parameters for mismatches next to labelled thymidine. (PDF)

S3 Table Evaluation of hairpin stability prediction. (PDF)

## References

[1] VanGuilder HD, Vrana KE, Freeman WM. Twenty-five years of quantitative PCR for gene expression analysis. Biotechniques. 2008;44: 619–26. 10.2144/000112776 18474036

[2] Morrison TB, Weis JJ, Wittwer CT. Quantification of low-copy transcripts by continuous SYBR Green I monitoring during amplification. Biotechniques. 1998;24: 954–8, 960, 962 Available: http://www.ncbi.nlm.nih.gov/pubmed/9631186 9631186

[3] Didenko V V. DNA probes using fluorescence resonance energy transfer (FRET): designs and applications. Biotechniques. 2001;31: 1106–16, 1118, 1120–1. Available: http://www.pubmedcentral.nih.gov/articlerender.fcgi?artid=1941713&tool=pmcentrez&rendertype=abstract 11730017 10.2144/01315rv02 PMC1941713

[4] Holland PM, Abramson RD, Watson R, Gelfand DH. Detection of specific polymerase chain reaction product by utilizing the 5’——3' exonuclease activity of Thermus aquaticus DNA polymerase. Proc Natl Acad Sci U S A. 1991;88: 7276–80. Available: http://www.pubmedcentral.nih.gov/articlerender.fcgi?artid=52277&tool=pmcentrez&rendertype=abstract 1871133 10.1073/pnas.88.16.7276 PMC52277

[5] Tyagi S, Kramer FR. Molecular beacons: probes that fluoresce upon hybridization. Nat Biotechnol. 1996;14: 303–8. 10.1038/nbt0396-303 9630890

[6] Caplin BE, Rasmussen RP, Bernard PS, Wittwer CT, Pathology E, City SL. LightCyclerTM Hybridization Probes The most direct way to monitor PCR amplification for quantification and mutation detection. BIOCHEMICA. 1999; 2–5.

[7] Rozen S, Skaletsky H. Primer3 on the WWW for general users and for biologist programmers. Methods Mol Biol. 2000;132: 365–86. Available: http://www.ncbi.nlm.nih.gov/pubmed/10547847 10547847 10.1385/1-59259-192-2:365

[8] Untergasser A, Cutcutache I, Koressaar T, Ye J, Faircloth BC, Remm M, et al Primer3—new capabilities and interfaces. Nucleic Acids Res. 2012;40: e115 10.1093/nar/gks596 22730293 PMC3424584

[9] Marshall OJ. PerlPrimer: cross-platform, graphical primer design for standard, bisulphite and real-time PCR. Bioinformatics. 2004;20: 2471–2. 10.1093/bioinformatics/bth254 15073005

[10] SantaLucia J. Physical principles and visual-OMP software for optimal PCR design. Methods Mol Biol. 2007;402: 3–34. 10.1007/978-1-59745-528-2_1 17951788

[11] Mann T, Humbert R, Dorschner M, Stamatoyannopoulos J, Noble WS. A thermodynamic approach to PCR primer design. Nucleic Acids Res. 2009;37: e95 10.1093/nar/gkp443 19528077 PMC2715258

[12] Ye J, Coulouris G, Zaretskaya I, Cutcutache I, Rozen S, Madden TL. Primer-BLAST: a tool to design target-specific primers for polymerase chain reaction. BMC Bioinformatics. 2012;13: 134 10.1186/1471-2105-13-134 22708584 PMC3412702

[13] Álvarez-Fernández R. Explanatory chapter: PCR primer design. Methods Enzymol. 2013;529: 1–21. 10.1016/B978-0-12-418687-3.00001-X 24011032

[14] Wittwer CT. High-resolution DNA melting analysis: advancements and limitations. Hum Mutat. 2009;30: 857–9. 10.1002/humu.20951 19479960

[15] Liu Y-P, Wu H-Y, Yang X, Xu H-Q, Chen D, Huang Q, et al Diagnostic accuracy of high resolution melting analysis for detection of KRAS mutations: a systematic review and meta-analysis. Sci Rep. 2014;4: 7521 10.1038/srep07521 25515911 PMC4268648

[16] Vossen RHAM, Aten E, Roos A, den Dunnen JT. High-resolution melting analysis (HRMA): more than just sequence variant screening. Hum Mutat. 2009;30: 860–6. 10.1002/humu.21019 19418555

[17] Hanami T, Delobel D, Kanamori H, Tanaka Y, Kimura Y, Nakasone A, et al Eprobe Mediated Real-Time PCR Monitoring and Melting Curve Analysis. Lin B, editor. PLoS One. 2013;8: e70942 10.1371/journal.pone.0070942 23951046 PMC3737233

[18] Atsumi J, Hanami T, Enokida Y, Ogawa H, Delobel D, Mitani Y, et al Eprobe-mediated screening system for somatic mutations in the KRAS locus. Oncol Rep. 2015;33: 2719–27. 10.3892/or.2015.3883 25823645 PMC4431451

[19] Ikeda S, Okamoto A. Hybridization-sensitive on-off DNA probe: application of the exciton coupling effect to effective fluorescence quenching. Chem Asian J. 2008;3: 958–68. 10.1002/asia.200800014 18446920

[20] Okamoto A. ECHO probes: a concept of fluorescence control for practical nucleic acid sensing. Chem Soc Rev. 2011;40: 5815–28. 10.1039/c1cs15025a 21660343

[21] Ikeda S, Kubota T, Yuki M, Okamoto A. Exciton-controlled hybridization-sensitive fluorescent probes: multicolor detection of nucleic acids. Angew Chem Int Ed Engl. 2009;48: 6480–4. 10.1002/anie.200902000 19637175

[22] Kimura Y, Hanami T, Tanaka Y, de Hoon MJL, Soma T, Harbers M, et al Effect of Thiazole Orange Doubly Labeled Thymidine on DNA Duplex Formation. Biochemistry. 2012;51: 6056–6067. 10.1021/bi300293d 22765348

[23] R Development Core Team. R: A language and environment for statistical computing. [Internet]. Vienna, Austria: R Foundation for Statistical Computing; 2011. Available: http://www.r-project.org/.

[24] Markham NR, Zuker M. UNAFold : Software for Nucleic Acid Folding and Hybridization Keith JM, editor. Bioinformatics, Vol II Struct Funct Appl vol 453. Totowa, NJ: Humana Press; 2008;453. 10.1007/978-1-60327-429-6_1 18712296

[25] Kierzek R, Burkard ME, Turner DH. Thermodynamics of single mismatches in RNA duplexes. Biochemistry. 1999;38: 14214–23. 10571995 10.1021/bi991186l

[26] Davis AR, Znosko BM. Positional and neighboring base pair effects on the thermodynamic stability of RNA single mismatches. Biochemistry. 2010;49: 8669–79. 10.1021/bi100146z 20681613 PMC2991393

[27] Naiser T, Kayser J, Mai T, Michel W, Ott A. Position dependent mismatch discrimination on DNA microarrays–experiments and model. BMC Bioinformatics. 2008;9: 509 10.1186/1471-2105-9-509 19046422 PMC2661940

[28] SantaLucia J. A unified view of polymer, dumbbell, and oligonucleotide DNA nearest-neighbor thermodynamics. Proc Natl Acad Sci U S A. 1998;95: 1460–5. Available: http://www.pubmedcentral.nih.gov/articlerender.fcgi?artid=19045&tool=pmcentrez&rendertype=abstract 9465037 10.1073/pnas.95.4.1460 PMC19045

[29] Ikeda S, Kubota T, Kino K, Okamoto A. Sequence dependence of fluorescence emission and quenching of doubly thiazole orange labeled DNA: effective design of a hybridization-sensitive probe. Bioconjug Chem. 2008;19: 1719–25. 10.1021/bc800201m 18666792

[30] Lee LG, Chen CH, Chiu L a. Thiazole orange: a new dye for reticulocyte analysis. Cytometry. 1986;7: 508–17. 10.1002/cyto.990070603 2430763

[31] Spielmann HP, Wemmer DE, Jacobsen JP. Solution structure of a DNA complex with the fluorescent bis-intercalator TOTO determined by NMR spectroscopy. Biochemistry. 1995;34: 8542–53. Available: http://www.ncbi.nlm.nih.gov/pubmed/9576807 7612596 10.1021/bi00027a004

[32] Sugizaki K, Okamoto A. ECHO-LNA conjugates: hybridization-sensitive fluorescence and its application to fluorescent detection of various RNA strands. Bioconjug Chem. 2010;21: 2276–81. 10.1021/bc1002949 21090641

[33] SantaLucia J, Hicks D. The thermodynamics of DNA structural motifs. Annu Rev Biophys Biomol Struct. 2004;33: 415–40. 10.1146/annurev.biophys.32.110601.141800 15139820

[34] McTigue PM, Peterson RJ, Kahn JD. Sequence-dependent thermodynamic parameters for locked nucleic acid (LNA)-DNA duplex formation. Biochemistry. 2004;43: 5388–405. 10.1021/bi035976d 15122905

