# Correction: Edesign: Primer and Enhanced Internal Probe Design Tool for Quantitative PCR Experiments and Genotyping Assays

**DOI:** 10.1371/journal.pone.0152713

**Published:** 2016-03-24

**Authors:** The PLOS ONE Staff

The Supporting Information file S1 Table is incomplete. The publisher apologizes for the error. Please see the complete S1 Table file here.

## Supporting Information

S1 Table New Functions in Edesign. (PDF)
