# Supplementary material for: Edesign: Primer and Enhanced Internal Probe Design Tool for Quantitative PCR Experiments and Genotyping Assays
Source: PLoS One. 2016 Feb 10;11(2):e0146950. doi: 10.1371/journal.pone.0146950 (PMC4749234; doi:10.1371/journal.pone.0146950)
Supplement: S1 Fig — (PDF) [file pone.0146950.s002.pdf]

A. Overall structure of Edesign core program

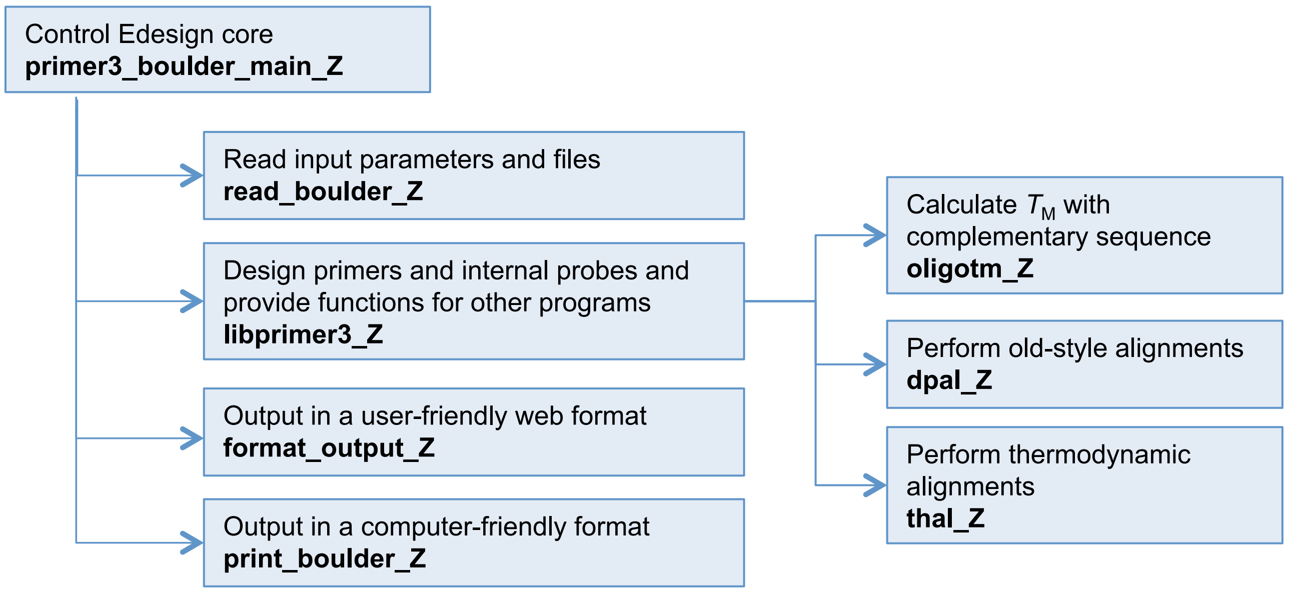

B. Workflow of primer and internal probe design in libprimer3_Z

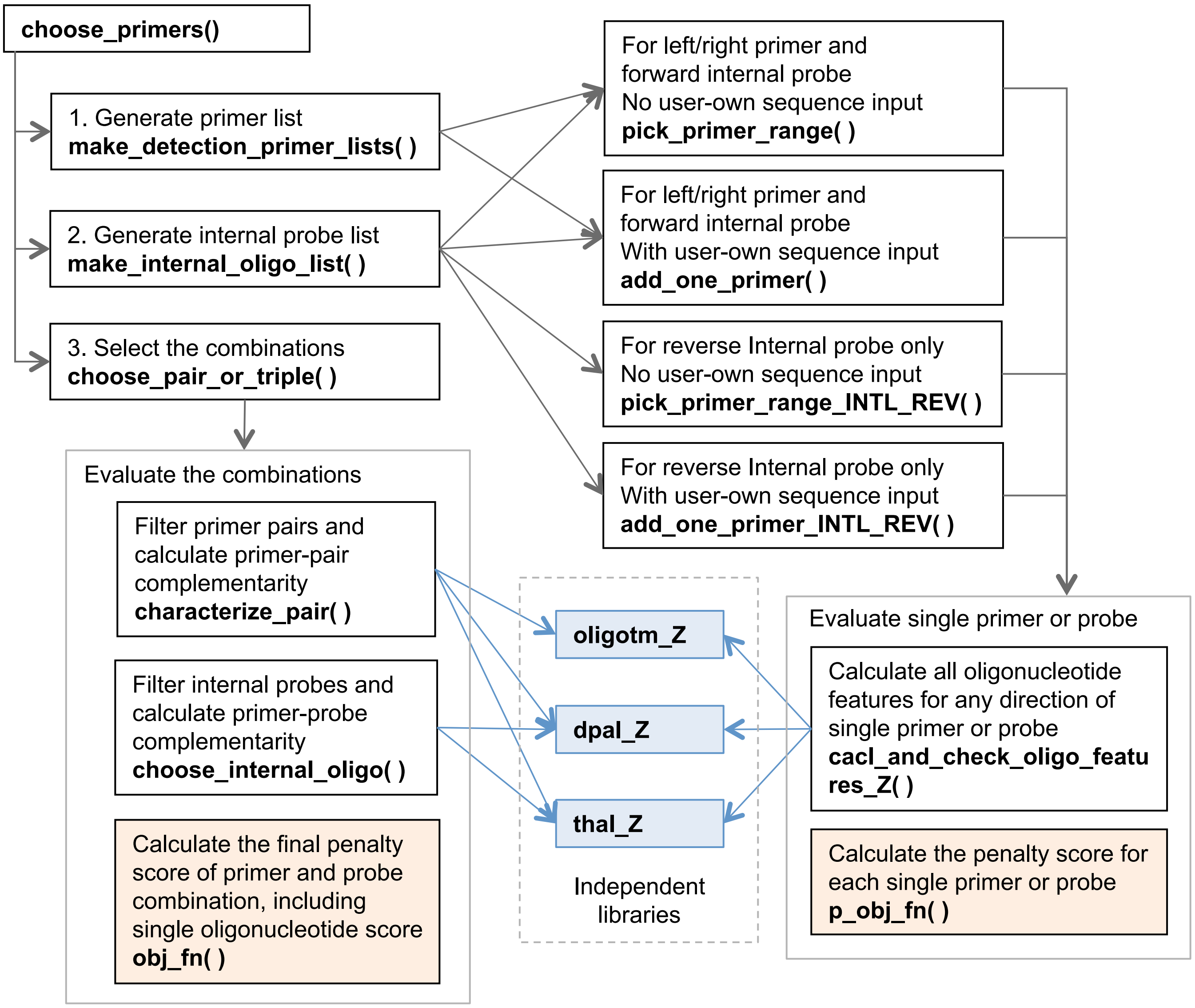

S1 Fig. Schematic structure of Edesign core program.
(A) Overall structure of Edesign core program. (B) Workflow of primer and internal probe design run by choose_primers() in libprimer3_Z.
