# Supplementary material for: Edesign: Primer and Enhanced Internal Probe Design Tool for Quantitative PCR Experiments and Genotyping Assays
Source: PLoS One. 2016 Feb 10;11(2):e0146950. doi: 10.1371/journal.pone.0146950 (PMC4749234; doi:10.1371/journal.pone.0146950)
Supplement: S2 Fig — (PDF) [file pone.0146950.s003.pdf]

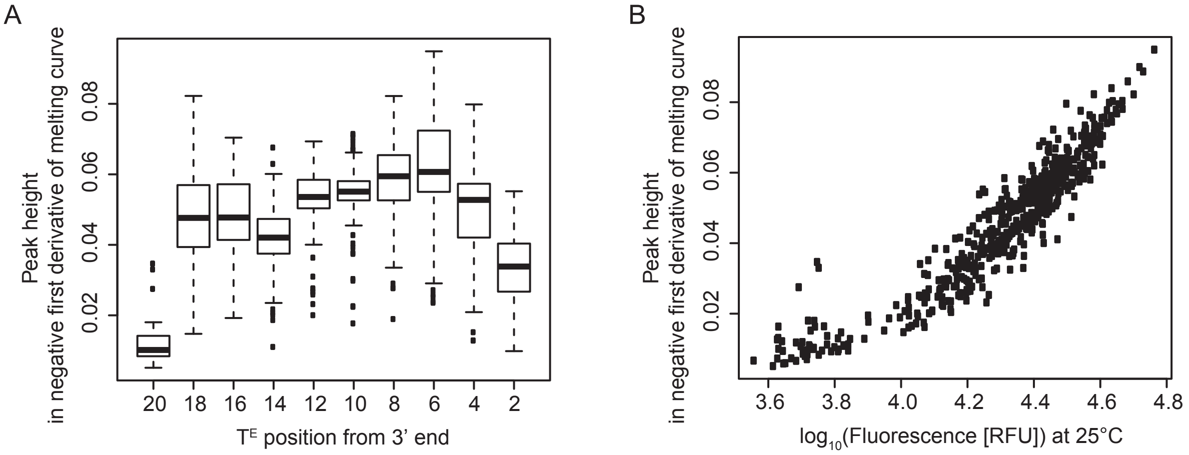

S2 Fig. Summary on peak heights of negative first derivative of melting curves.
(A) Boxplot for the peak height of negative first derivative of melting curves at each of the labelling positions. Each boxplot summarizes fluorescent melting curve experiments of one ECHO with 61 complementary oligonucleotides (one full-match and 60 single-base mismatch sequences), using mean values among replicates of each ECHO/DNA pair.
(B) Plot of the log_{10} values of fluorescence at 25°C versus the height of negative first derivative of melting curve. All the combinations of the 20-mer ECHO/DNA hybrids in this study were used. Mean values among replicates were plotted.
