# Supplementary material for: Edesign: Primer and Enhanced Internal Probe Design Tool for Quantitative PCR Experiments and Genotyping Assays
Source: PLoS One. 2016 Feb 10;11(2):e0146950. doi: 10.1371/journal.pone.0146950 (PMC4749234; doi:10.1371/journal.pone.0146950)
Supplement: S3 Fig — (PDF) [file pone.0146950.s004.pdf]

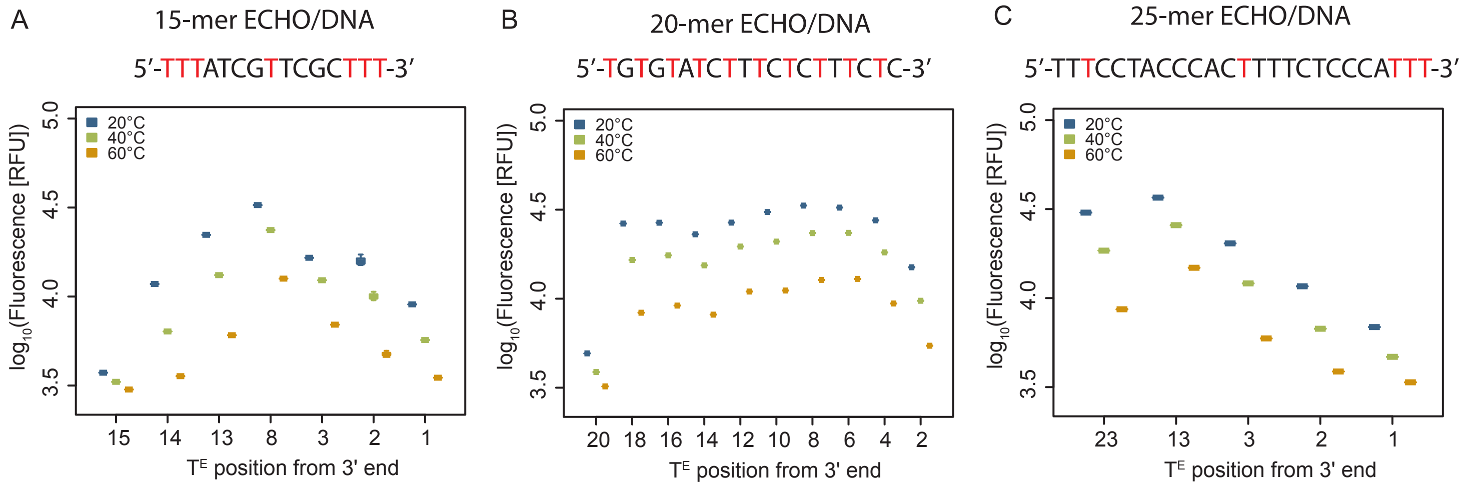

S3 Fig. Fluorescence signals of 15-mer, 20-mer and 25-mer ECHOs.
Fluorescence signals observed in melting curves of ECHO/DNA duplexes of 15-mer (A), 20-mer (B) and 25-mer (C). ECHO sequences used are shown in the top of each plot. Red-colored thymidines are the examined labelling positions. 20-mer sequences are same as described in the main text. 15-mer and 25-mer sequences were designed in the same way as for 20-mer described in the main text. All the antisense DNA oligonucleotides used were full-matched sequences of the ECHOs. All replicates were used to draw each boxplot. The first, second and third bases from the 5’ and 3’ terminal of ECHOs were mainly examined.
