# Supplementary material for: Edesign: Primer and Enhanced Internal Probe Design Tool for Quantitative PCR Experiments and Genotyping Assays
Source: PLoS One. 2016 Feb 10;11(2):e0146950. doi: 10.1371/journal.pone.0146950 (PMC4749234; doi:10.1371/journal.pone.0146950)
Supplement: S4 Fig — (PDF) [file pone.0146950.s005.pdf]

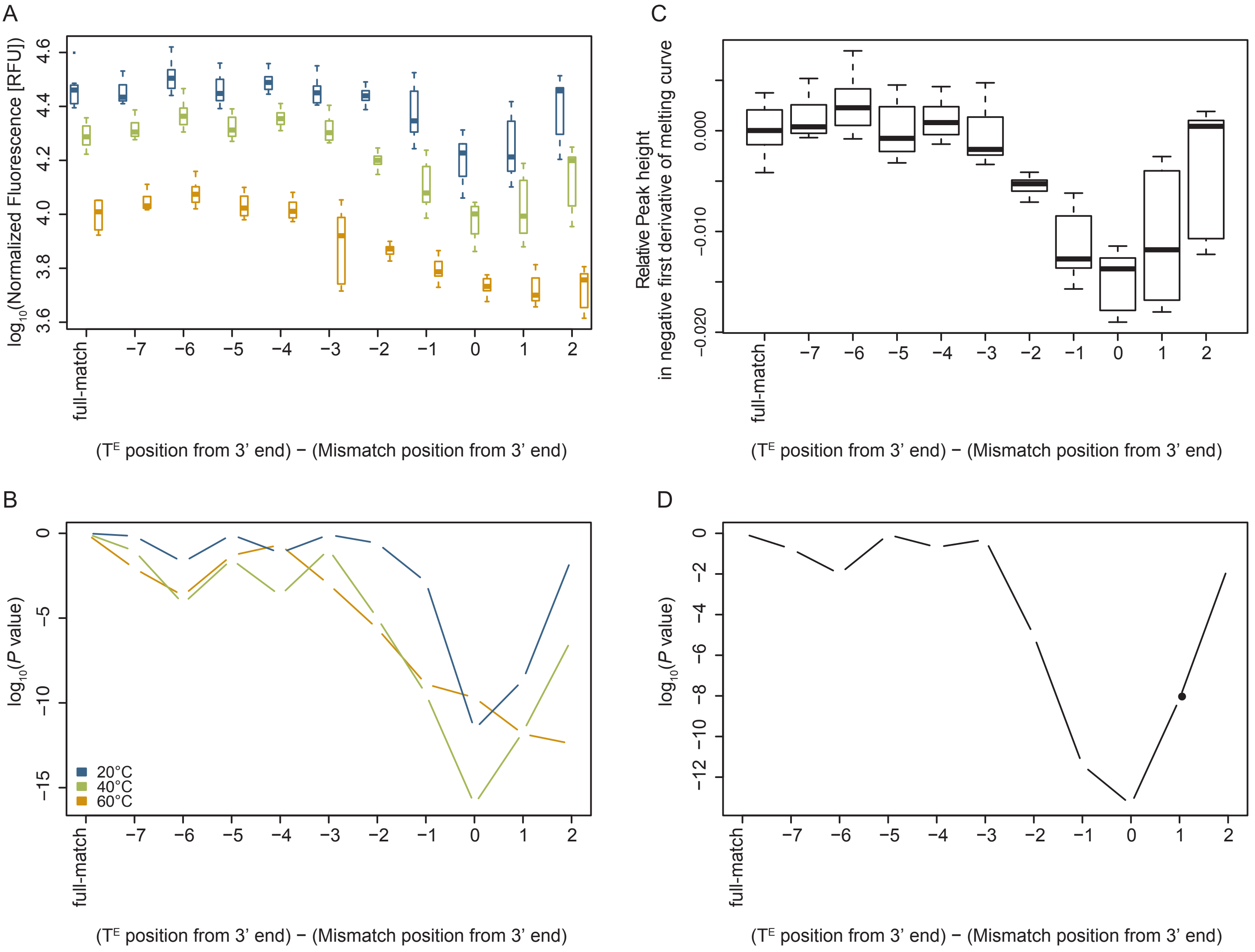

S4 Fig. Effect of mismatched base pair positions relative to the thiazole-orange labelled nucleotide in 15-mer ECHO/DNA hybrids.

Effect of mismatched base pair positions relative to the thiazole-orange labelled nucleotide on (A) fluorescence intensity (at 20, 40 and 60°C) and its *P* value (B) by comparison to full-match ECHO/DNA hybrids (Student’s T test, two-tailed), and (C) peak height in negative first derivative of melting curves and its *P* value (D) by comparison to full-match ECHO/DNA hybrids (Student’s T test, two-tailed).

For this analysis, two 15-mer ECHOs, 5’-TTT^{E}ATCGTTCGCTTT-3’, 5’-TTTATCGT^{E}TCGCTTT-3’ and anti-sense oligonucleotides that contains single mismatches at position 11, 12, 13, 14 and 15 from 3’ end of the ECHOs were used. In total 15 different antisense oligonucleoties and 30 ECHO/DNA combinations were applied for conducting meting curve experiments. The fluorescence intensities were normalized between two ECHOs using the fluorescence intensities of fully-matched ECHO/DNAs. The peak height in negative first derivative of melting curves were analyzed using relative heights from the height of fully-matched ECHO/DNAs. Each boxplot and point in (A-D) summarizes ECHO/DNA pairs with different ECHOs having the same distance from the labelled nucleotide to a mismatch, using all replicates.
