# Supplementary material for: Edesign: Primer and Enhanced Internal Probe Design Tool for Quantitative PCR Experiments and Genotyping Assays
Source: PLoS One. 2016 Feb 10;11(2):e0146950. doi: 10.1371/journal.pone.0146950 (PMC4749234; doi:10.1371/journal.pone.0146950)
Supplement: S5 Fig — (PDF) [file pone.0146950.s006.pdf]

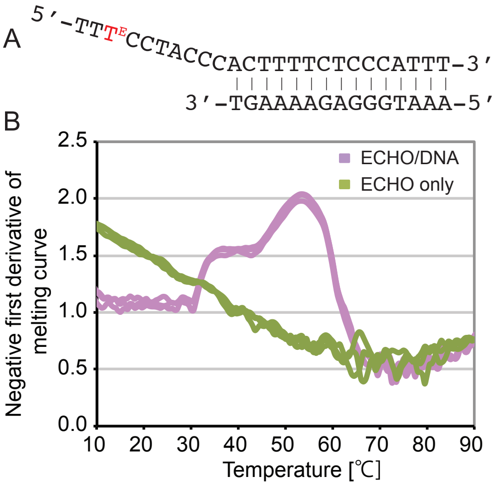

S5 Fig. Fluorescent signal of dye moiety located outside of duplex region. Partially-complementary ECHO (labelled base indicated as red-colored T^E) and DNA oligonucleotide shown in (A) were prepared. These two oligonucleotides form a hybrid where the labelled thymine is 7 nucleotides away from the double-stranded region. For fluoresence melting curve assay, ECHO and DNA oligonucleotides were dissolved in a buffer containing 1.4mM dNTP, 20mM Tris-HCl, 10mM $(NH_4)_2SO_4$, 8mM $MgSO_4$, 0.1% Tween-20, 10mM KCl at a concentration of 1 μM. Fluoresence melting curves were measured by the same method as described in the main text. The negative first derivative of the melting curve of this ECHO/DNA duplex (B) showed a melting temperature around 53 °C, which is a little higher than predicted melting temperature of 50°C for the DNA/DNA duplex of the underlined sequence.
