# Supplementary material for: Edesign: Primer and Enhanced Internal Probe Design Tool for Quantitative PCR Experiments and Genotyping Assays
Source: PLoS One. 2016 Feb 10;11(2):e0146950. doi: 10.1371/journal.pone.0146950 (PMC4749234; doi:10.1371/journal.pone.0146950)
Supplement: S6 Fig — (PDF) [file pone.0146950.s007.pdf]

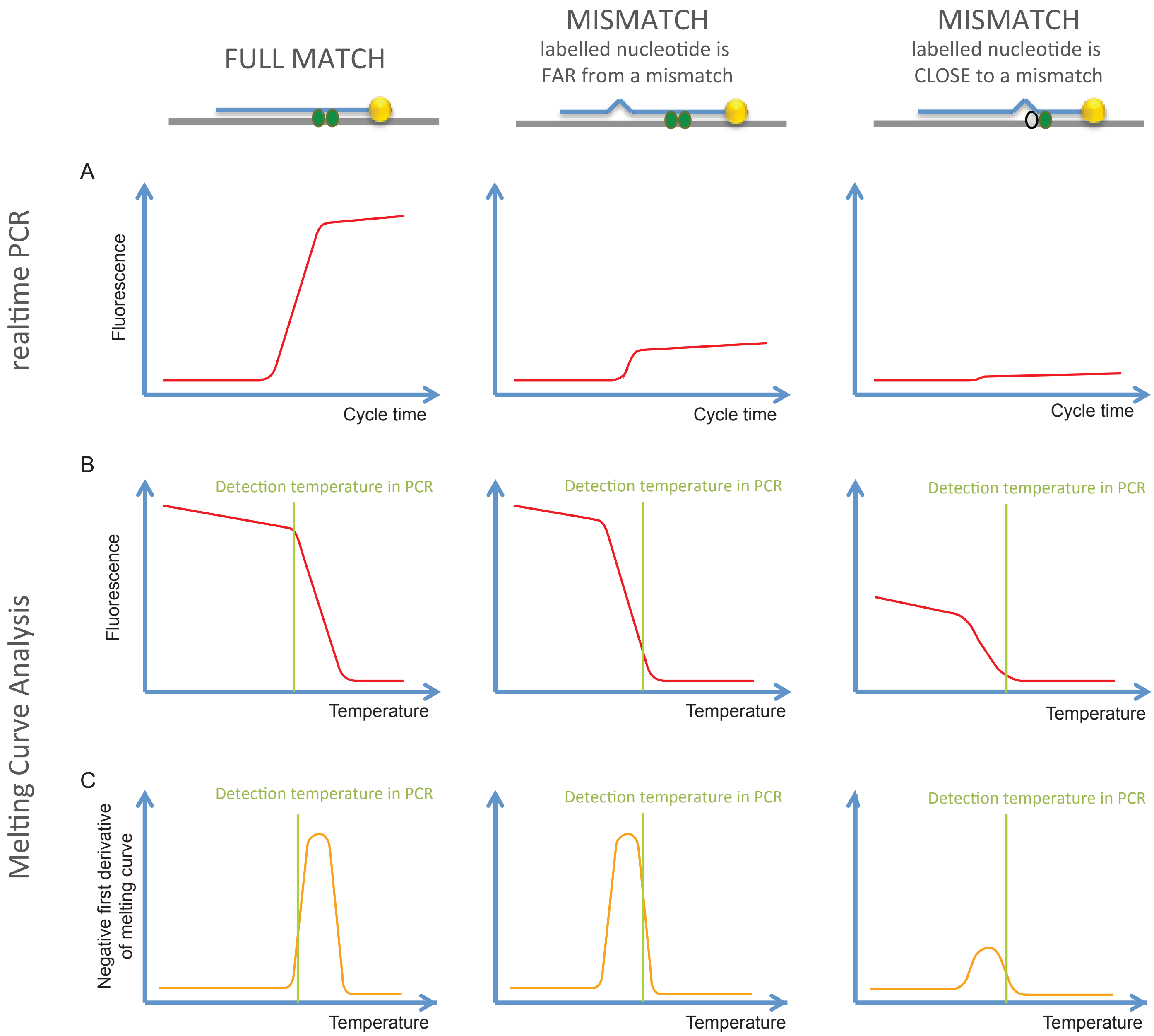

S6 Fig. Interpretation of Eprobe mediated PCR and melting curve experiments.
Conceptual drawings of fluorescence signals from Eprobes during real-time PCR (A) and melting curve experiments (B, C) are shown for Eprobes: (left) fully-matching template, (middle), mismatch with template where a mismatch is located more than two nucleotides away from a labelled nucleotide, and (right) mismatch with template where a mismatch is located within one nucleotide from a labelled nucleotide.
During the amplification reaction the fluorescent signal of the Eprobe is only detected at one temperature defined in the settings of the PCR instrument (A; The detection temperature for the signals shown in A are indicated as “Detection temperature in PCR” in B and C). This is not a problem when working with an Eprobe having a perfect match with its template, where the T_M value can be selected accordingly. However, the signal strength during real-time monitoring can be reduced if the Eprobe does not have the same melting temperature for the wildtype and mutant type. In cases where the T_M values for binding to a mismatch fall below the temperature used for the signal detection, the signal will be reduced because it depends on the ratio of free to bound Eprobe. The extend of the reduction depends on where the mismatch is located. As indicated in the figure, labelling positions around the mismatch can dramatically reduce the signal up to the point that hardly any signal will be obtained. A setting that greatly reduces the signal is suitable when the user conducts genotyping only by the fluorescent signal in real-time PCR reaction (loss of function experiment).
The shift in the T_M values for the wildtype and mutant type will be recorded during melting curve analysis when the PCR instrument will measure the fluorescent signal over the entire temperature range given in the settings (B, C).
Taking negative first derivative of the melting curve (C), the Eprobe will provide the same level of peak height for the wildtype and mutant type if the mismatch is located more than two nucleotides away from the labelling position. Therefore in Edesign, under default settings the labelled nucleotide will be placed more than two nucleotides away from the mismatch. However, the user has the option to place the mismatch around the labelling position, which will lead to a reduced signal or even to a loss of signal. Therefore this position is not suitable to conduct melting curve experiments, and should only be used for real-time PCR experiments as described above.
