# Supplementary material for: Edesign: Primer and Enhanced Internal Probe Design Tool for Quantitative PCR Experiments and Genotyping Assays
Source: PLoS One. 2016 Feb 10;11(2):e0146950. doi: 10.1371/journal.pone.0146950 (PMC4749234; doi:10.1371/journal.pone.0146950)
Supplement: S7 Fig — (PDF) [file pone.0146950.s008.pdf]

A. Primer and Eprobe design without Edesign function (values shown were re-calculated by Edesign.)

OLIGO	start	len	tm	tm(VAR)	gc%	any	3'	hairpin	seq
LEFT Primer	158	23	57.02	57.02	43.48	2.80	2.40	27.58	GGGAAAGATAGTCAAATCAGTCG
RIGHT Primer	350	20	58.91	58.91	55.00	3.00	2.40	0.00	GTCTCCGAAAATCCCACTGC
Eprobe (Reverse)	215	17	58.39	55.92	41.18	5.40	3.40	54.32	GCATTCCZCATAGTGAT

product size: 193,
Primer pair compl any: 4.40, 3': 3.20,
LEFT Primer-Probe compl any: 4.20, 3': 1.60,
RIGHT Primer-Probe compl any: 4.00, 3': 2.00

Location of designed primers and Eprobe

```
        X                             X     X
  1 ACAACGGCATAATAACAGACACTATCAAGAGTTGGAGAAACAATATATTGAGAACACAAG
        x                             x     x

     X  X                       X  X     XX X                  X
 61 AGTCTGAATGTGCATGTGTAAATGGTTCTTGCTTTACTGTAATGACCGATGGACCAAGTG
     x  x                       x  x     xx x                  x

           X                   X         >>>>>>>>>>>>>>>>>>>>>>>
121 ATGGACAGGCCTCATACAAGATCTTCAGAATAGAAAAGGGAAAGATAGTCAAATCAGTCG
           x                   x

     X
181 AAATGAATGCCCCTAATTATCACTATGAGGAATGCTCCTGTTATCCTGATTCTAGTGAAA
     x                ^^*^^^^^^^^^^^^^^

              X                     X                X
241 TCACATGTGTGTGCAGGGATAACTGGCATGGCTCGAATCGACCGTGGGTGTCTTTCAACC
              x                     x                x

                   X      X       <<<<<<<<<<<<<<<<<<<<
301 AGAATCTGGAATATCAGATAGGATACATATGCAGTGGGATTTTCGGAGACAATCCACGCC
                   x      x

           X    X                     X
361 CTAATGATAAGACAGGCAGTTGTGGTCCAGTATCGTCTAAT
           x    x                     x

XXXXXX excluded region
xxxxxx excluded region for internal probe
****** TARGET VARIANT
>>>>>> left primer
<<<<<< right primer
^^^^^^ internal probe
```

B. Primer and Eprobe design by Edesign

OLIGO	start	len	tm	tm(VAR)	gc%	any	3'	hairpin	seq
LEFT Primer	156	25	58.89	58.89	40.00	2.80	2.40	27.58	AAGGGAAAGATAGTCAAATCAGTCG
RIGHT Primer	366	21	58.79	58.79	52.38	2.40	0.00	1.81	CATTAGGGCGTGGATTGTCTC
Eprobe (Reverse)	204	21	61.72	56.41	38.10	7.00	0.60	0.00	AGTGATAATZAGGGGCATTCA

product size: 211,
Primer pair compl any: 3.80, 3': 1.20,
LEFT Primer-Probe compl any: 3.60, 3': 0.20,
RIGHT Primer-Probe compl any: 3.60, 3': 2.40

Location of designed primers and Eprobe

```
        X                             X     X
  1 ACAACGGCATAATAACAGACACTATCAAGAGTTGGAGAAACAATATATTGAGAACACAAG
        x                             x     x

     X  X                       X  X     XX X                  X
 61 AGTCTGAATGTGCATGTGTAAATGGTTCTTGCTTTACTGTAATGACCGATGGACCAAGTG
     x  x                       x  x     xx x                  x

           X                   X       >>>>>>>>>>>>>>>>>>>>>>>>>
121 ATGGACAGGCCTCATACAAGATCTTCAGAATAGAAAAGGGAAAGATAGTCAAATCAGTCG
           x                   x

     X
181 AAATGAATGCCCCTAATTATCACTATGAGGAATGCTCCTGTTATCCTGATTCTAGTGAAA
     x ^^^^^^^^^^^^^^^^^*^^^

              X                     X                X
241 TCACATGTGTGTGCAGGGATAACTGGCATGGCTCGAATCGACCGTGGGTGTCTTTCAACC
              x                     x                x

                   X      X                      <<<<<<<<<<<<<<<
301 AGAATCTGGAATATCAGATAGGATACATATGCAGTGGGATTTTCGGAGACAATCCACGCC
                   x      x

    <<<<<< X    X                     X
361 CTAATGATAAGACAGGCAGTTGTGGTCCAGTATCGTCTAAT
           x    x                     x

XXXXXX excluded region
xxxxxx excluded region for internal probe
****** TARGET VARIANT
>>>>>> left primer
<<<<<< right primer
^^^^^^ internal probe
```

S7 Fig. Designed Eprobes and primers for evaluation in mutation detection.
Design-A was conducted without any upgrade implemented in Edesign other than T_M calculation of Eprobe/DNA duplex. Design-B was conducted by Edesign. Design-A has higher predicted hairpin T_M of Eprobe and higher pair complementarity than Design-B.

Target sequence: pandemic 2009 H1N1 influenza virus genome segment 6 (neuraminidase) H275Y mutation

ACAACGGCATAATAACAGACACTATCAAGAGTTGGAGAAACAATATATTGAGAACACAAGAGTCTGAATGTGCATG
TGTAAATGGTTCTTGCTTTACTGTAATGACCGATGGACCAAGTGATGGACAGGCCTCATACAAGATCTTCAGAATA
GAAAAGGGAAAGATAGTCAAATCAGTCGAAATGAATGCCCCTAATTAT[C/T]ACTATGAGGAATGCTCCTGTTATCC
TGATTCTAGTGAAATCACATGTGTGTGCAGGGATAACTGGCATGGCTCGAATCGACCGTGGGTGTCTTTCAACCA
GAATCTGGAATATCAGATAGGATACATATGCAGTGGGATTTTCGGAGACAATCCACGCCCTAATGATAAGACAGGC
AGTTGTGGTCCAGTATCGTCTAAT

Edesign setting:

Positions below were set as excluded positions (non-target mutations) for primers and internal Eprobe:

5, 35, 41, 62, 65, 89, 92, 98, 99, 101, 120, 128, 148, 182, 251, 273, 290, 316, 323, 368, 373, 395

Genotyping by Internal Probe: ON

PRIMER_PRODUCT_SIZE_RANGE: 150-250 100-300 301-400 401-500 501-600 601-700 701-850 851-1000

Primer Size Min: 18 Opt: 20 Max: 25

TH: Primer Max Hairpin: 47 (No primers found with the default value 24)

Internal Probe Size Min: 16 Opt: 18 Max: 21

Internal Probe T_M Min: 57.0, Opt: 62.0, Max: 67.0

Weight of Internal Probe T_M Difference by Target Variant: 1.0

Weight of OLD: Pair 3' Complementarity: 1.0

Other parameter values were the same as the default settings of Edesign version 2.0.

Experimental Protocol:

PCR reactions were setup using 5 µl of 5×Light-Cycler 480 Genotyping Master (with 5% Formamide), 5 µl template DNA, 0.2 µM Eprobe, 0.9 µM primer in opposite strand of Eprobe and 0.3 µM primer in the same strand of Eprobe, in a total volume of 25 µl. Real-time PCR experiments were run on a LightCycler 480 (Roche Diagnostics, Mannheim, Germany) after activation of the hot-start enzyme for 10 min at 95°C, followed by 50 cycles of 15 s at 95°C, 30 s at 57°C, and 30 s at 72°C.

Amplification signals were detected during the annealing step of each cycle at 57°C, using a SYBR Green I (483 nm–533 nm) filter for thiazole orange (D514). For melting curve analysis, the PCR was followed by heating the reaction mixture to 95°C for 15 s, cooling to 37°C, holding at 37°C for 7 min, and then slowly heating again to 95°C at a ramp rate 2.2°C/s and continuous fluorescence acquisition at the indicated wave length. All PCR reactions and melting curve experiments were always performed in triplicate, and each experiment included a negative control where 1×TE Buffer (Promega Japan, Tokyo, Japan) was added instead of a template DNA.
