# Supplementary material for: Edesign: Primer and Enhanced Internal Probe Design Tool for Quantitative PCR Experiments and Genotyping Assays
Source: PLoS One. 2016 Feb 10;11(2):e0146950. doi: 10.1371/journal.pone.0146950 (PMC4749234; doi:10.1371/journal.pone.0146950)
Supplement: S8 Fig — (PDF) [file pone.0146950.s009.pdf]

A. Primer and Eprobe design without Edesign function (values shown were re-calculated by Edesign.)

OLIGO	start	len	tm	gc%	any	3'	hairpin	seq
LEFT Primer	76	18	59.20	61.11	5.40	3.40	34.23	GGCCATGAAAGCTCAGCG
RIGHT Primer	316	22	58.99	50.00	2.40	0.00	0.00	CGTCTTCTCCTTTTCCCATTCC
Eprobe (Forward)	190	16	58.92	50.00	3.00	1.80	4.97	TCACATZCACACAGGG

product size: 241,
Primer pair compl any: 3.60, 3': 1.20,
LEFT Primer-Probe compl any: 3.80, 3': 1.80,
RIGHT Primer-Probe compl any: 3.60, 3': 2.40

Location of designed primers and Eprobe

```
          X  X        X  X     X          X            X
   1 AAGAAAGGCCTGATTCTGGCTGAGAGAAAAATGAGAAGATGTGTGAGCTTTCATGAAGCA
          x  x        x  x     x          x            x

          X        X>>>>>>>>>>>>>>>>>>X    X  X     X
  61 TTTGAAATAGCAGAAGGCCATGAAAGCTCAGCGCTACTATACTGTCTCATGGTCATGTAC
          x        x                  x    x  x     x

                                              X           X  X  X
 121 CTGAATCCTGGAAATTATTCAATGCAAGTAAAACTAGGAACGCTCTGTGCTTTATGCGAG
                                              x           x  x  x

                                             XX     X     X  X
 181 AAACAAGCATCACATTCACACAGGGCTCATAGCAGAGCAGCGAGATCTTCAGTGCCTGGA
              ^^^^^^^^^^^^^^^^               xx     x     x  x

          X  X        X                                    <<<<<<
 241 GTGAGACGAGAAATGCAGATGGTCTCAGCTATGAACACAGCAAAAACAATGAATGGAATG
          x  x        x

     <<<<<<<<<<<<<<<< X     X     X     XX X                 XX
 301 GGAAAAGGAGAAGACGTCCAAAAGCTGGCAGAAGAGCTGCAAAGCAACATTGGAGTGCTG
                      x     x     x     xx x                 xx

                      X                 X
 361 AGATCTCTTGGGGCAAGTCAAAAGAATGGGGAAGGGATTGC
                      x                 x
```

```
XXXXXX excluded region
xxxxxx excluded region for internal probe
>>>>>> left primer
<<<<<< right primer
^^^^^^ internal probe
```

B. Primer and Eprobe design by Edesign

OLIGO	start	len	tm	gc%	any	3'	hairpin	seq
LEFT Primer	190	20	59.24	50.00	2.80	0.80	0.02	TCACATTCACACAGGGCTCA
RIGHT Primer	316	22	58.99	50.00	2.40	0.00	0.00	CGTCTTCTCCTTTTCCCATTCC
Eprobe (Reverse)	288	16	58.66	37.50	2.40	0.40	0.00	TGTTTTTGCZGTGTTC

product size: 127,
Primer pair compl any: 3.60, 3': 0.00,
LEFT Primer-Probe compl any: 6.00, 3': 1.40,
RIGHT Primer-Probe compl any: 2.20, 3': 0.00

Location of designed primers and Eprobe

```
          X  X        X  X     X          X            X
   1 AAGAAAGGCCTGATTCTGGCTGAGAGAAAAATGAGAAGATGTGTGAGCTTTCATGAAGCA
          x  x        x  x     x          x            x

          X        X                  X    X  X     X
  61 TTTGAAATAGCAGAAGGCCATGAAAGCTCAGCGCTACTATACTGTCTCATGGTCATGTAC
          x        x                  x    x  x     x

                                              X           X  X  X
 121 CTGAATCCTGGAAATTATTCAATGCAAGTAAAACTAGGAACGCTCTGTGCTTTATGCGAG
                                              x           x  x  x

              >>>>>>>>>>>>>>>>>>>>           XX     X     X  X
 181 AAACAAGCATCACATTCACACAGGGCTCATAGCAGAGCAGCGAGATCTTCAGTGCCTGGA
                                             xx     x     x  x

          X  X        X                                    <<<<<<
 241 GTGAGACGAGAAATGCAGATGGTCTCAGCTATGAACACAGCAAAAACAATGAATGGAATG
          x  x        x              ^^^^^^^^^^^^^^^^

     <<<<<<<<<<<<<<<< X     X     X     XX X                 XX
 301 GGAAAAGGAGAAGACGTCCAAAAGCTGGCAGAAGAGCTGCAAAGCAACATTGGAGTGCTG
                      x     x     x     xx x                 xx

                      X                 X
 361 AGATCTCTTGGGGCAAGTCAAAAGAATGGGGAAGGGATTGC
                      x                 x
```

```
XXXXXX excluded region
xxxxxx excluded region for internal probe
>>>>>> left primer
<<<<<< right primer
^^^^^^ internal probe
```

S8 Fig. Designed Eprobes and primers for evaluation in low copy detection.
Design-A was conducted without any upgrade implemented in Edesign. Design-B was conducted by Edesign. Design-A has higher pair complementarity especially PRIMER-Eprobe complementarity than Design-B.

Target sequence: influenza B virus genome segment 7 (matrix protein)

AAGAAAGGCCTGATTCTGGCTGAGAGAAAAATGAGAAGATGTGTGAGCTTTCATGAAGCATTTGAAATAGCAGAAG
GCCATGAAAGCTCAGCGCTACTATACTGTCTCATGGTCATGTACCTGAATCCTGGAAATTATTCAATGCAAGTAAA
ACTAGGAACGCTCTGTGCTTTATGCGAGAAACAAGCATCACATTCACACAGGGCTCATAGCAGAGCAGCGAGATC
TTCAGTGCCTGGAGTGAGACGAGAAATGCAGATGGTCTCAGCTATGAACACAGCAAAAACAATGAATGGAATGGG
AAAAGGAGAAGACGTCCAAAAGCTGGCAGAAGAGCTGCAAAGCAACATTGGAGTGCTGAGATCTCTTGGGGCAA
GTCAAAAGAATGGGGAAGGGATTGC

Edesign setting:

Positions below were set as “excluded positions” for primers and internal Eprobe in Edesign because of their high mutation rates in the virus:

6, 9, 18, 21, 27, 38, 51, 66, 75, 94, 99, 102, 108, 162, 174, 177, 180, 221, 222, 228, 234, 237, 246, 249, 258, 318, 324, 330, 336, 337, 339, 357, 358, 378, 396

Internal Probe Size Min: 14 Opt: 16 Max: 18

Internal Probe T_M Min: 54.0, Opt: 59.0, Max: 64.0

PRIMER_PRODUCT_SIZE_RANGE: 150-250 100-300 301-400 401-500 501-600 601-700 701-850 851-1000

Weight of OLD: Pair 3' Complementarity: 1.0

Other parameter values were the same as the default settings of Edesign version 2.0.

Experimental Protocol:

PCR reactions were setup using 5 µl of 5×Light-Cycler 480 Genotyping Master (with 5% Formamide), 5 µl template DNA, 0.2 µM Eprobe, 0.9 µM primer in opposite strand of Eprobe and 0.3 µM primer in the same strand of Eprobe, in a total volume of 25 µl. Real-time PCR experiments were run on a LightCycler 480 (Roche Diagnostics, Mannheim, Germany) after activation of the hot-start enzyme for 10 min at 95°C, followed by 50 cycles of 15 s at 95°C, 30 s at 57°C, and 30 s at 72°C.

Amplification signals were detected during the annealing step of each cycle at 57°C, using a SYBR Green I (483 nm–533 nm) filter for thiazole orange (D514). For melting curve analysis, the PCR was followed by heating the reaction mixture to 95°C for 15 s, cooling to 37°C, holding at 37°C for 7 min, and then slowly heating again to 95°C at a ramp rate 2.2°C/s and continuous fluorescence acquisition at the indicated wave length. All PCR reactions and melting curve experiments were always performed in triplicate, and each experiment included a negative control where 1×TE Buffer (Promega Japan, Tokyo, Japan) was added instead of a template DNA.

The template concentration per reaction varied from 150 to 150,000,000 copies of plasmid DNA.
