# Supplementary material for: Edesign: Primer and Enhanced Internal Probe Design Tool for Quantitative PCR Experiments and Genotyping Assays
Source: PLoS One. 2016 Feb 10;11(2):e0146950. doi: 10.1371/journal.pone.0146950 (PMC4749234; doi:10.1371/journal.pone.0146950)
Supplement: S1 Table — (PDF) [file pone.0146950.s010.pdf]

S1 Table. New Functions in Edesign.

Category	New Function	Name in Web interface	Description	modified CORE programs
Enhanced internal probe design	Internal probe design on any direction	Internal Probe Direction	Strand of internal probe can be selected from "Forward", "Reverse", "Any". "Any" will list candidate internal probe from both strands. When a user specifies own probe sequence, the user must specify "Forward" or Reverse".	read_boulder_Z.c<br>libprimer3_Z.h<br>libprimer3_Z.c
	Multiple Internal probe selection	-	Primer3 returns only one best internal probe for each of primer pair. Upgraded Edesign selection procedure returns multiple internal probes if their scores are high.	libprimer3_Z.h<br>libprimer3_Z.c
	Internal probe mishybridization check	OLD: Internal Probe Max Template Mishyb	Edesign calculates mishybridization of internal probe in the target sequence (binding to sequences exept for intentional binding site).	read_boulder_Z.c<br>libprimer3_Z.h<br>libprimer3_Z.c
		TH: Internal Probe Template Mishyb		
	Primer-internal probe complement check	OLD: Pair Max Any Complementarity	Edesign includes complement check of Primer bound to Internal probe in Primer Pair complementary check.	libprimer3_Z.h<br>libprimer3_Z.c
		OLD: Pair Max 3' Complementarity		
		TH: Pair Max Any Complementarity		
		TH: Pair Max 3' Complementarity		
Genotyping mode	Genotyping mode ON/OFF	Genotyping by Internal Probe	If this is checked, Edesign will attempt to prick internal probe to detect the target variant. Internal probe site is restricted to overlap the target variant. Target variant in the target sequence must be specified using "[", "]" and "/". Different variants are separated by "/".	primer3_boulder_main_Z.c<br>read_boulder_Z.c<br>libprimer3_Z.h<br>libprimer3_Z.c
	Setting for T_M against target variant<br><br>(In the genotyping mode, Edesign calculates the melting temperatures of internal probes against Target Variant sequence)	Internal Probe Tm Difference by Target Variant	Penalty weight for the difference of the melting temperatures of an internal probe caused by the Target Variant. Larger the difference, it is easier to discriminate a mismatch case from a perfect match case. If this weight is larger than 0, higher penalty is given to smaller T_M difference.	read_boulder_Z.c<br>libprimer3_Z.h<br>libprimer3_Z.c
		Internal Probe Tm Min for Target Variant	Edesign will NOT pick internal probes whose melting temparature against the target variant are smaller than this parameter.	read_boulder_Z.c<br>libprimer3_Z.h<br>libprimer3_Z.c
	Displaying T_M for target variant	tm(VAR)	T_M of the primer or probe against the target variant sequence is displayed.	libprimer3_Z.h<br>libprimer3_Z.c<br>format_output_Z.c
Treating modified oligonucleotides<br><br>Modification (ECHO)-specific features	Internal probe modification ON/OFF	Set Internal Probe as Eprobe	If this is checked, Edesign conducts design of primers and probes with modified nucleotide "Z".	read_boulder_Z.c<br>libprimer3_Z.h<br>libprimer3_Z.c<br>format_output_Z.c<br>print_boulder_Z.c
	Left primer modification ON/OFF	Set Left primer as Eprimer		
	Rright primer modification ON/OFF	Set Right primer as Eprimer		
	Set modifiable nucleotide	-	Modifiable nucleotide ("A", "C", "G" or "T" converted to "Z") can be set for a custom Edesign	modification_Z.h
	Calculation of TM with modified nucleotide	Primer Tm	TM of oligomer is calculated with modified-nucleotide thermodynamics (ECHO/DNA thermodynamics)	oligotm_Z.c
		Internal Probe Tm		
	Calculation of oligomer complementarity with modified nucleotide	OLD: Primer Max Template Mispriming	When "Use Thermodynamic Alignment" is NOT checked (default), these parameters are calculated with scores:<br>match of A or T: 0.8<br>match of G or C: 1.2<br>match of Z (labelled T): 3.0<br>match of N: 0.25<br>mismatch: -1.0	dpal_Z.c
		OLD: Pair Max Template Mispriming		
		OLD: Primer Max Self Complementarity		
		OLD: Primer Max 3' Self Complementarity		
		OLD: Pair Max Any Complementarity		
		OLD: Pair Max 3' Complementarity		
		OLD: Internal Probe Max Template Mishyb		
		OLD: Internal Probe Max Self Complementarity		
		OLD: Internal Probe Max 3' Self Complementarity		
		TH: Primer Max Template Mispriming	When "Use Thermodynamic Alignment" is checked, these parameters are calculated with modified-nucleotide thermodynamics (ECHO/DNA thermodynamics).	thal_Z.c<br>primer3_config_Z/stack.dh<br>primer3_config_Z/stack.ds<br>primer3_config_Z/stackmm.dh<br>primer3_config_Z/stackmm.ds<br>primer3_config_Z/tstack.dh<br>primer3_config_Z/tstack2.dh<br>primer3_config_Z/tstack2.ds<br>primer3_config_Z/tstack_tm_inf.ds
		TH: Pair Max Template Mispriming		
		TH: Primer Max Self Complementarity		
		TH: Primer Max 3' Self Complementarity		
		TH: Pair Max Any Complementarity		
		TH: Pair Max 3' Complementarity		
		TH: Internal Probe Max Template Mishyb		
		TH: Internal Probe Max Self Complementarity		
		TH: Internal Probe Max 3' Self Complementarity		
		TH: Primer Max Hairpin	Melting temperature of hairpin is calculated with modified-nucleotide thermodynamics (ECHO/DNA thermodynamics).	
		TH: Internal Probe Max Hairpin		
	Setting for modified nucleotide position	Primer 5' Terminal Region Excluded for Labelling	Edesign will examine all possible modification positions other than nucleotides specified by these parameters.<br><br>(Inner nucleotides of Eprimer/Eprobe are preferable to be labelled. Labelling 3' side nucleotides of Eprimer is also preferable since it will be inside after extension by polymerase.)	read_boulder_Z.c<br>libprimer3_Z.h<br>libprimer3_Z.c
		Primer 3' Terminal Region Excluded for Labelling		
		Internal Probe 5' Terminal Region Excluded for Labelling		
		Internal Probe 3' Terminal Region Excluded for Labelling		
		Labelling Terminal Side	Penalty weight for modification positions next to the excluded region in case those positions are possible for modification but not a best option.	read_boulder_Z.c<br>libprimer3_Z.h<br>libprimer3_Z.c
	Setting for modified nucleotide position for Genotyping	Internal Probe Labelling Position against Target Variant	If this is NOT checked (default), a modified nucleotide "Z" will be designed far from the target variant more than specified nucleotides (the default is 2: "Z" will be on 3, 4 or further nucleotide from the target variant.).<br><br>If this is CHECKED, the modified nucleotide "Z" is designed near or on the target variant within specified nucleotides (the default is 1: "Z" will be on 0 or 1 nucleotides from the target variant.).	read_boulder_Z.c<br>libprimer3_Z.h<br>libprimer3_Z.c
