# Supplementary material for: Edesign: Primer and Enhanced Internal Probe Design Tool for Quantitative PCR Experiments and Genotyping Assays
Source: PLoS One. 2016 Feb 10;11(2):e0146950. doi: 10.1371/journal.pone.0146950 (PMC4749234; doi:10.1371/journal.pone.0146950)
Supplement: S2 Table — (PDF) [file pone.0146950.s011.pdf]

## S2 Table. Thermodynamic parameters used for nearest neighbor parameters for mismatch next to labelled thymidine.

| | | | | | Observed thermodynamic parameters for ECHO/DNA | | | | |
|---|---|---|---|---|---|---|---|---|---|
| No | Mismatch type | Trinucleotide (5' to 3' / 5' to 3') | ECHO sequence (5' to 3) | Antisense DNA sequence (5' to 3') | $\Delta H°$ [kcal mol$^{-1}$] | $\Delta S°$ [cal mol$^{-1}$ K$^{-1}$] | $\Delta G°_{37}$ [kcal mol$^{-1}$] | $\Delta G°_{60}$ [kcal mol$^{-1}$] | $T_M$ [°C] |
| 1 | E16/17_A | GZA/TAA | TGTGZATCTTTCTCTTTCTC | GAGAAAGAGAAAGATAAACA | -137.6 ± 0.86 | -375.6 ± 2.53 | -21.1 ± 0.07 | -12.5 ± 0.02 | 65.9 ± 0.03 |
| 2 | E16/17_G | GZA/TAG | TGTGZATCTTTCTCTTTCTC | GAGAAAGAGAAAGATAGACA | -137.2 ± 1.07 | -373.8 ± 3.19 | -21.3 ± 0.09 | -12.7 ± 0.03 | 66.4 ± 0.07 |
| 3 | E16/17_T | GZA/TAT | TGTGZATCTTTCTCTTTCTC | GAGAAAGAGAAAGATATACA | -146.8 ± 3.65 | -401.9 ± 10.79 | -22.1 ± 0.31 | -12.9 ± 0.08 | 66.5 ± 0.14 |
| 4 | E14/15_A | AZC/GAA | TGTGTAZCTTTCTCTTTCTC | GAGAAAGAGAAAGAAACACA | -124.9 ± 0.79 | -337.9 ± 2.28 | -20.1 ± 0.08 | -12.4 ± 0.04 | 66.3 ± 0.07 |
| 5 | E14/15_C | AZC/GAC | TGTGTAZCTTTCTCTTTCTC | GAGAAAGAGAAAGACACACA | -120.2 ± 1.82 | -323.0 ± 5.31 | -20.1 ± 0.17 | -12.6 ± 0.06 | 67.3 ± 0.09 |
| 6 | E14/15_G | AZC/GAG | TGTGTAZCTTTCTCTTTCTC | GAGAAAGAGAAAGAGACACA | -127.6 ± 2.22 | -343.7 ± 6.35 | -21.0 ± 0.26 | -13.1 ± 0.12 | 68.0 ± 0.23 |
| 7 | E12/13_A | CZT/AAA | TGTGTATCZTTCTCTTTCTC | GAGAAAGAGAAAAATACACA | -125.9 ± 0.24 | -343.3 ± 0.75 | -19.4 ± 0.01 | -11.5 ± 0.00 | 63.8 ± 0.02 |
| 8 | E12/13_C | CZT/AAC | TGTGTATCZTTCTCTTTCTC | GAGAAAGAGAAACATACACA | -104.7 ± 9.15 | -282.1 ± 26.68 | -17.2 ± 0.87 | -10.7 ± 0.26 | 61.9 ± 0.69 |
| 9 | E12/13_T | CZT/AAT | TGTGTATCZTTCTCTTTCTC | GAGAAAGAGAAATATACACA | -111.2 ± 8.35 | -301.6 ± 24.36 | -17.7 ± 0.79 | -10.7 ± 0.23 | 61.9 ± 0.60 |
| 10 | E10/11_C | TZC/GAC | TGTGTATCTTZCTCTTTCTC | GAGAAAGAGACAGATACACA | -127.2 ± 2.40 | -344.9 ± 6.79 | -20.2 ± 0.31 | -12.3 ± 0.16 | 65.9 ± 0.36 |
| 11 | E10/11_G | TZC/GAG | TGTGTATCTTZCTCTTTCTC | GAGAAAGAGAGAGATACACA | -134.8 ± 1.88 | -364.7 ± 5.25 | -21.7 ± 0.25 | -13.3 ± 0.14 | 68.1 ± 0.24 |
| 12 | E10/11_T | TZC/GAT | TGTGTATCTTZCTCTTTCTC | GAGAAAGAGATAGATACACA | -134.0 ± 2.03 | -364.2 ± 6.02 | -21.1 ± 0.18 | -12.7 ± 0.08 | 66.7 ± 0.19 |
| 13 | E18/17_A | GZG/AAC | TGZGTATCTTTCTCTTTCTC | GAGAAAGAGAAAGATAAACA | -152.9 ± 7.43 | -421.6 ± 21.61 | -22.2 ± 0.73 | -12.5 ± 0.24 | 65.4 ± 0.29 |
| 14 | E18/17_G | GZG/GAC | TGZGTATCTTTCTCTTTCTC | GAGAAAGAGAAAGATAGACA | -145.1 ± 0.69 | -398.6 ± 1.98 | -21.5 ± 0.08 | -12.3 ± 0.04 | 65.3 ± 0.09 |
| 15 | E18/17_T | GZG/TAC | TGZGTATCTTTCTCTTTCTC | GAGAAAGAGAAAGATATACA | -166.8 ± 14.36 | -461.0 ± 41.97 | -23.9 ± 1.34 | -13.2 ± 0.38 | 66.5 ± 0.22 |
| 16 | E16/15_A | GZA/AAC | TGTGZATCTTTCTCTTTCTC | GAGAAAGAGAAAGAAACACA | -123.3 ± 1.36 | -333.2 ± 4.03 | -20.0 ± 0.11 | -12.3 ± 0.04 | 66.1 ± 0.10 |
| 17 | E16/15_C | GZA/CAC | TGTGZATCTTTCTCTTTCTC | GAGAAAGAGAAAGACACACA | -113.4 ± 0.61 | -302.6 ± 1.93 | -19.6 ± 0.08 | -12.6 ± 0.08 | 67.7 ± 0.27 |
| 18 | E16/15_G | GZA/GAC | TGTGZATCTTTCTCTTTCTC | GAGAAAGAGAAAGAGACACA | -131.3 ± 1.29 | -354.8 ± 3.78 | -21.3 ± 0.13 | -13.1 ± 0.05 | 68.0 ± 0.09 |
| 19 | E14/13_A | AZC/AAT | TGTGTAZCTTTCTCTTTCTC | GAGAAAGAGAAAAATACACA | -116.8 ± 11.20 | -318.0 ± 32.63 | -18.2 ± 1.08 | -10.8 ± 0.33 | 62.2 ± 0.79 |
| 20 | E14/13_C | AZC/CAT | TGTGTAZCTTTCTCTTTCTC | GAGAAAGAGAAACATACACA | -90.1 ± 0.00 | -237.4 ± 0.00 | -16.4 ± 0.00 | -11.0 ± 0.00 | 63.4 ± 0.00 |
| 21 | E14/13_T | AZC/TAT | TGTGTAZCTTTCTCTTTCTC | GAGAAAGAGAAATATACACA | -121.2 ± 4.59 | -329.4 ± 13.62 | -19.1 ± 0.37 | -11.5 ± 0.05 | 63.9 ± 0.00 |
| 22 | E12/11_C | CZT/CAG | TGTGTATCZTTCTCTTTCTC | GAGAAAGAGACAGATACACA | -111.3 ± 1.70 | -297.5 ± 5.17 | -19.0 ± 0.10 | -12.2 ± 0.03 | 66.5 ± 0.17 |
| 23 | E12/11_G | CZT/GAG | TGTGTATCZTTCTCTTTCTC | GAGAAAGAGAGAGATACACA | -129.8 ± 1.99 | -351.4 ± 5.80 | -20.8 ± 0.20 | -12.7 ± 0.08 | 67.0 ± 0.14 |
| 24 | E12/11_T | CZT/TAG | TGTGTATCZTTCTCTTTCTC | GAGAAAGAGATAGATACACA | -125.9 ± 1.62 | -339.8 ± 4.74 | -20.5 ± 0.15 | -12.7 ± 0.05 | 67.2 ± 0.09 |

| | | Thermodynamic parameters of normal DNA | | | | | Positional effect | | | |
|---|---|---|---|---|---|---|---|---|---|---|
| No | Mismatch type | $\Delta H°$ [kcal mol$^{-1}$] | $\Delta S°$ [cal mol$^{-1}$ K$^{-1}$] | $\Delta G°_{37}$ [kcal mol$^{-1}$] | $\Delta G°_{60}$ [kcal mol$^{-1}$] | $\Delta T_M$ [°C] | $\Delta\Delta H°$ [kcal mol$^{-1}$] | $\Delta\Delta S°$ [cal mol$^{-1}$ K$^{-1}$] | $\Delta\Delta G°_{37}$ [kcal mol$^{-1}$] | $\Delta\Delta G°_{60}$ [kcal mol$^{-1}$] |
| 1 | E16/17_A | -134.80 | -379.46 | -17.11 | -8.38 | 55.91 | 17.3 ± 4.66 | 52.3 ± 13.55 | 1.1 ± 0.49 | -0.1 ± 0.23 |
| 2 | E16/17_G | -139.10 | -391.49 | -17.68 | -8.68 | 56.72 | 19.6 ± 4.39 | 58.7 ± 12.64 | 1.4 ± 0.53 | 0.1 ± 0.31 |
| 3 | E16/17_T | -139.80 | -394.36 | -17.49 | -8.42 | 56.14 | 20.7 ± 3.52 | 62.8 ± 10.03 | 1.3 ± 0.46 | -0.2 ± 0.29 |
| 4 | E14/15_A | -133.90 | -373.30 | -18.12 | -9.53 | 58.70 | 11.7 ± 9.41 | 31.2 ± 28.10 | 2.0 ± 0.72 | 1.3 ± 0.20 |
| 5 | E14/15_C | -131.00 | -365.21 | -17.73 | -9.33 | 58.16 | 11.2 ± 4.75 | 30.0 ± 14.02 | 1.9 ± 0.46 | 1.2 ± 0.23 |
| 6 | E14/15_G | -139.70 | -389.23 | -18.98 | -10.03 | 59.93 | 15.8 ± 7.79 | 45.5 ± 23.20 | 1.6 ± 0.64 | 0.6 ± 0.24 |
| 7 | E12/13_A | -128.20 | -361.47 | -16.09 | -7.78 | 54.17 | 5.2 ± 8.06 | 13.3 ± 24.19 | 1.1 ± 0.65 | 0.8 ± 0.35 |
| 8 | E12/13_C | -132.00 | -374.27 | -15.92 | -7.31 | 53.21 | 19.3 ± 19.12 | 55.1 ± 56.84 | 2.2 ± 1.55 | 1.0 ± 0.48 |
| 9 | E12/13_T | -138.10 | -391.75 | -16.60 | -7.59 | 54.15 | 10.7 ± 11.03 | 31.7 ± 33.00 | 0.9 ± 0.85 | 0.2 ± 0.31 |
| 10 | E10/11_C | -136.60 | -384.04 | -17.49 | -8.66 | 56.62 | 8.3 ± 8.71 | 24.3 ± 25.66 | 0.8 ± 0.78 | 0.2 ± 0.27 |
| 11 | E10/11_G | -138.60 | -389.39 | -17.83 | -8.87 | 57.18 | 12.1 ± 6.97 | 36.3 ± 20.92 | 0.8 ± 0.55 | 0.0 ± 0.26 |
| 12 | E10/11_T | -140.80 | -397.52 | -17.51 | -8.37 | 56.05 | 13.9 ± 9.39 | 42.5 ± 27.79 | 0.7 ± 0.80 | -0.3 ± 0.26 |
| 13 | E18/17_A | -134.80 | -379.46 | -17.11 | -8.38 | 55.91 | 17.3 ± 4.66 | 52.3 ± 13.55 | 1.1 ± 0.49 | -0.1 ± 0.23 |
| 14 | E18/17_G | -139.10 | -391.49 | -17.68 | -8.68 | 56.72 | 19.6 ± 4.39 | 58.7 ± 12.64 | 1.4 ± 0.53 | 0.1 ± 0.31 |
| 15 | E18/17_T | -139.80 | -394.36 | -17.49 | -8.42 | 56.14 | 20.7 ± 3.52 | 62.8 ± 10.03 | 1.3 ± 0.46 | -0.2 ± 0.29 |
| 16 | E16/15_A | -133.90 | -373.30 | -18.12 | -9.53 | 58.70 | 11.7 ± 9.41 | 31.2 ± 28.10 | 2.0 ± 0.72 | 1.3 ± 0.20 |
| 17 | E16/15_C | -131.00 | -365.21 | -17.73 | -9.33 | 58.16 | 11.2 ± 4.75 | 30.0 ± 14.02 | 1.9 ± 0.46 | 1.2 ± 0.23 |
| 18 | E16/15_G | -139.70 | -389.23 | -18.98 | -10.03 | 59.93 | 15.8 ± 7.79 | 45.5 ± 23.20 | 1.6 ± 0.64 | 0.6 ± 0.24 |
| 19 | E14/13_A | -128.20 | -361.47 | -16.09 | -7.78 | 54.17 | 5.2 ± 8.06 | 13.3 ± 24.19 | 1.1 ± 0.65 | 0.8 ± 0.35 |
| 20 | E14/13_C | -132.00 | -374.27 | -15.92 | -7.31 | 53.21 | 19.3 ± 19.12 | 55.1 ± 56.84 | 2.2 ± 1.55 | 1.0 ± 0.48 |
| 21 | E14/13_T | -138.10 | -391.75 | -16.60 | -7.59 | 54.15 | 10.7 ± 11.03 | 31.7 ± 33.00 | 0.9 ± 0.85 | 0.2 ± 0.31 |
| 22 | E12/11_C | -136.60 | -384.04 | -17.49 | -8.66 | 56.62 | 8.3 ± 8.71 | 24.3 ± 25.66 | 0.8 ± 0.78 | 0.2 ± 0.27 |
| 23 | E12/11_G | -138.60 | -389.39 | -17.83 | -8.87 | 57.18 | 12.1 ± 6.97 | 36.3 ± 20.92 | 0.8 ± 0.55 | 0.0 ± 0.26 |
| 24 | E12/11_T | -140.80 | -397.52 | -17.51 | -8.37 | 56.05 | 13.9 ± 9.39 | 42.5 ± 27.79 | 0.7 ± 0.80 | -0.3 ± 0.26 |

| | | Nearest Neighbor parameters of full-match | | | | | Nearest Neighbor parameters with mismatch | | | | |
|---|---|---|---|---|---|---|---|---|---|---|---|
| No | Mismatch type | Nearest neighbor (5' to 3' / 5' to 3') | $\Delta\Delta H°$ [kcal mol$^{-1}$] | $\Delta\Delta S°$ [cal mol$^{-1}$ K$^{-1}$] | $\Delta\Delta G°_{37}$ [kcal mol$^{-1}$] | $\Delta\Delta G°_{60}$ [kcal mol$^{-1}$] | Nearest neighbor (5' to 3' / 5' to 3') | $\Delta\Delta H°$ [kcal mol$^{-1}$] | $\Delta\Delta S°$ [cal mol$^{-1}$ K$^{-1}$] | $\Delta\Delta G°_{37}$ [kcal mol$^{-1}$] | $\Delta\Delta G°_{60}$ [kcal mol$^{-1}$] |
| 1 | E16/17_A | ZA/TA | -8.9 ± 0.73 | -20.7 ± 1.26 | -2.4 ± 0.20 | -1.9 ± 0.10 | GZ/AA | -11.2 ± 4.79 | -27.7 ± 13.84 | -2.6 ± 0.53 | -2.0 ± 0.25 |
| 2 | E16/17_G | ZA/TA | -8.9 ± 0.73 | -20.7 ± 1.26 | -2.4 ± 0.20 | -1.9 ± 0.10 | GZ/AG | -8.9 ± 4.58 | -20.2 ± 13.10 | -2.6 ± 0.57 | -2.1 ± 0.33 |
| 3 | E16/17_T | ZA/TA | -8.9 ± 0.73 | -20.7 ± 1.26 | -2.4 ± 0.20 | -1.9 ± 0.10 | GZ/AT | -18.9 ± 5.13 | -49.7 ± 14.79 | -3.5 ± 0.60 | -2.3 ± 0.32 |
| 4 | E14/15_A | ZC/GA | -8.9 ± 0.73 | -21.3 ± 1.25 | -2.3 ± 0.22 | -1.8 ± 0.14 | AZ/AA | 6.2 ± 9.47 | 25.4 ± 28.22 | -1.7 ± 0.76 | -2.3 ± 0.24 |
| 5 | E14/15_C | ZC/GA | -8.9 ± 0.73 | -21.3 ± 1.25 | -2.3 ± 0.22 | -1.8 ± 0.14 | AZ/AC | 8.5 ± 5.14 | 33.6 ± 15.04 | -1.9 ± 0.54 | -2.7 ± 0.28 |
| 6 | E14/15_G | ZC/GA | -8.9 ± 0.73 | -21.3 ± 1.25 | -2.3 ± 0.22 | -1.8 ± 0.14 | AZ/AG | 5.3 ± 8.13 | 21.3 ± 24.08 | -1.3 ± 0.72 | -1.8 ± 0.30 |
| 7 | E12/13_A | ZT/AA | -1.2 ± 0.72 | 0.5 ± 1.24 | -1.3 ± 0.21 | -1.3 ± 0.11 | CZ/AA | -1.7 ± 8.10 | 4.4 ± 24.24 | -3.0 ± 0.68 | -3.1 ± 0.36 |
| 8 | E12/13_C | ZT/AA | -1.2 ± 0.72 | 0.5 ± 1.24 | -1.3 ± 0.21 | -1.3 ± 0.11 | CZ/AC | 9.2 ± 21.21 | 36.6 ± 62.80 | -2.1 ± 1.79 | -3.0 ± 0.56 |
| 9 | E12/13_T | ZT/AA | -1.2 ± 0.72 | 0.5 ± 1.24 | -1.3 ± 0.21 | -1.3 ± 0.11 | CZ/AT | 17.3 ± 13.85 | 58.0 ± 41.03 | -0.6 ± 1.18 | -2.0 ± 0.40 |
| 10 | E10/11_C | ZC/GA | -8.9 ± 0.73 | -21.3 ± 1.25 | -2.3 ± 0.22 | -1.8 ± 0.14 | TZ/AC | 10.0 ± 9.06 | 36.2 ± 26.58 | -1.2 ± 0.87 | -2.0 ± 0.35 |
| 11 | E10/11_G | ZC/GA | -8.9 ± 0.73 | -21.3 ± 1.25 | -2.3 ± 0.22 | -1.8 ± 0.14 | TZ/AG | 0.7 ± 7.26 | 9.7 ± 21.61 | -2.3 ± 0.64 | -2.6 ± 0.32 |
| 12 | E10/11_T | ZC/GA | -8.9 ± 0.73 | -21.3 ± 1.25 | -2.3 ± 0.22 | -1.8 ± 0.14 | TZ/AT | 1.8 ± 9.63 | 12.2 ± 28.46 | -2.0 ± 0.85 | -2.3 ± 0.31 |
| 13 | E18/17_A | GZ/AC | -3.9 ± 0.69 | -7.0 ± 1.18 | -1.8 ± 0.20 | -1.6 ± 0.11 | ZG/AA | -31.5 ± 8.80 | -87.4 ± 25.54 | -4.4 ± 0.90 | -2.4 ± 0.35 |
| 14 | E18/17_G | GZ/AC | -3.9 ± 0.69 | -7.0 ± 1.18 | -1.8 ± 0.20 | -1.6 ± 0.11 | ZG/GA | -21.7 ± 4.50 | -58.7 ± 12.85 | -3.5 ± 0.57 | -2.2 ± 0.33 |
| 15 | E18/17_T | GZ/AC | -3.9 ± 0.69 | -7.0 ± 1.18 | -1.8 ± 0.20 | -1.6 ± 0.11 | ZG/TA | -43.8 ± 14.80 | -122.4 ± 43.16 | -5.8 ± 1.44 | -3.0 ± 0.49 |
| 16 | E16/15_A | GZ/AC | -3.9 ± 0.69 | -7.0 ± 1.18 | -1.8 ± 0.20 | -1.6 ± 0.11 | ZA/AA | 2.9 ± 9.53 | 15.9 ± 28.42 | -2.0 ± 0.75 | -2.4 ± 0.23 |
| 17 | E16/15_C | GZ/AC | -3.9 ± 0.69 | -7.0 ± 1.18 | -1.8 ± 0.20 | -1.6 ± 0.11 | ZA/CA | 10.3 ± 4.84 | 39.6 ± 14.20 | -2.0 ± 0.51 | -2.9 ± 0.27 |
| 18 | E16/15_G | GZ/AC | -3.9 ± 0.69 | -7.0 ± 1.18 | -1.8 ± 0.20 | -1.6 ± 0.11 | ZA/GA | -3.4 ± 7.93 | -4.1 ± 23.53 | -2.2 ± 0.68 | -2.1 ± 0.27 |
| 19 | E14/13_A | AZ/AT | -6.9 ± 0.75 | -16.1 ± 1.30 | -1.9 ± 0.22 | -1.5 ± 0.10 | ZC/AA | 13.1 ± 13.82 | 46.2 ± 40.64 | -1.3 ± 1.28 | -2.3 ± 0.49 |
| 20 | E14/13_C | AZ/AT | -6.9 ± 0.75 | -16.1 ± 1.30 | -1.9 ± 0.22 | -1.5 ± 0.10 | ZC/CA | 29.5 ± 19.14 | 97.8 ± 56.85 | -0.8 ± 1.57 | -3.1 ± 0.49 |
| 21 | E14/13_T | AZ/AT | -6.9 ± 0.75 | -16.1 ± 1.30 | -1.9 ± 0.22 | -1.5 ± 0.10 | ZC/TA | 13.0 ± 11.97 | 46.7 ± 35.72 | -1.5 ± 0.95 | -2.6 ± 0.33 |
| 22 | E12/11_C | CZ/AG | -6.9 ± 0.58 | -13.4 ± 1.01 | -2.7 ± 0.18 | -2.4 ± 0.12 | ZT/CA | 23.9 ± 8.89 | 75.7 ± 26.20 | 0.4 ± 0.80 | -1.4 ± 0.30 |
| 23 | E12/11_G | CZ/AG | -6.9 ± 0.58 | -13.4 ± 1.01 | -2.7 ± 0.18 | -2.4 ± 0.12 | ZT/GA | 3.6 ± 7.28 | 15.2 ± 21.73 | -1.1 ± 0.61 | -1.5 ± 0.29 |
| 24 | E12/11_T | CZ/AG | -6.9 ± 0.58 | -13.4 ± 1.01 | -2.7 ± 0.18 | -2.4 ± 0.12 | ZT/TA | 7.9 ± 9.55 | 28.7 ± 28.21 | -1.0 ± 0.83 | -1.7 ± 0.29 |
