# Supplementary material for: Edesign: Primer and Enhanced Internal Probe Design Tool for Quantitative PCR Experiments and Genotyping Assays
Source: PLoS One. 2016 Feb 10;11(2):e0146950. doi: 10.1371/journal.pone.0146950 (PMC4749234; doi:10.1371/journal.pone.0146950)
Supplement: S3 Table — (PDF) [file pone.0146950.s012.pdf]

S3 Table. Evaluation of hairpin stability prediction.

Name	ECHO Sequence	DNA oligomer T_M [°C]	Observed T_M [°C]	Predicted T_M [°C]	T_M difference (Obs - Pre) [°C]
TE_SLF_1	ACTTTTTT^{E}GCATTAGCAAAT	67.3	78.3 ± 0.6	96.8	-18.5
TE_SLF_2	ACTTT^{E}TTTGCATTAGCAAAT	67.3	68.0 ± 0.9	68.2	-0.2
TE_SLF_3	ACTT^{E}TTTTGCATTAGCAAAT	67.3	80.4 ± 0.5	68.2	12.2
TE_SLF_4	ACT^{E}TTTTTGCATTAGCAAAT	67.3	77.8 ± 0.6	68.2	9.6
TE_SLF_5	ACTTTCGTTTT^{E}TTTAAACGT	65.2	73.6 ± 6.3	62.6	11.0
TE_SLF_6	ACTTTCGT^{E}TTTTTTAAACGT	65.2	79.0 ± 0.2	81.6	-2.6
TE_SLF_7	ACTTTCGTTTTTTT^{E}AAACGT	65.2	66.1 ± 5.4	62.6	3.5
TE_SLF_8	ACTTTCGTTTTTT^{E}TTAAACGT	65.2	77.0 ± 0.9	62.6	14.4
TE_SLF_9	TTTT^{E}ATACATCTATAAAA	49.5	62.4 ± 2.3	72.8	-10.4
TE_SLF_10	AGATTTT^{E}GGAGGAACCAAACTG	65.4	76.7 ± 0.1	93.1	-16.4
TE_SLF_11	AGATTTT^{E}GGCGGCCCCAAACTG	64.2	76.2 ± 0.6	95.5	-19.3
TE_SLF_12	AGATTTT^{E}CGGCGGCCCAAACTG	38.5	69.2 ± 3.2	75.4	-6.2

Summary of ECHO self-folding prediction. 'T^E's in ECHO sequences indicates the thiazole orange double-labeled thymidine, underlined sequences are presumable double-stranded stem regions. The observed T_M values were obtained in the same way as described in MATERIALS AND METHODS. Predicted T_M values were calculated by the updated thermodynamic alignment program, "thal_Z", under the salt condition used in the experiments (program parameters set to 1000.2 mM of Na^+ and 0 mM of Mg^{2+}). As a reference, DNA oligonucleotide T_M values were calculated in the same way for DNA sequences of the same compositions (replacing of T^E by T). The averaged difference between the observed and predicted T_M values was -1.9 ± 12.3 °C.
